# New Guinean orogenic dynamics and biota evolution revealed using a custom geospatial analysis pipeline

**DOI:** 10.1186/s12862-021-01764-2

**Published:** 2021-04-06

**Authors:** Emmanuel F. A. Toussaint, Lloyd T. White, Helena Shaverdo, Athena Lam, Suriani Surbakti, Rawati Panjaitan, Bob Sumoked, Thomas von Rintelen, Katayo Sagata, Michael Balke

**Affiliations:** 1 Natural History Museum of Geneva, CP 6434, 1211 Geneva 6, Switzerland; 2 GeoQuEST Research Centre, School of Earth, Atmospheric and Life Sciences, University of Wollongong, Wollongong, NSW 2522 Australia; 3 Naturhistorisches Museum Wien, Burgring 7, 1010 Vienna, Austria; 4 SNSB‐Zoologische Staatssammlung München, Munich, Germany; 5 Department of Environmental Science, Policy and Management, University of California, Berkeley, CA USA; 6 Institute for Biodiversity Science and Sustainability, California Academy of Sciences, San Francisco, CA USA; 7 Department of Biology, Universitas Cenderawasih (UNCEN), Waena, Papua Indonesia; 8 Department of Biology, Faculty of Sciences and Mathematics, State University of Papua (UNIPA), Jalan Gunung Salju Amban, Manokwari, 98314 West Papua Indonesia; 9 Walian 2, Tomohon Selatan, 95439 N Sulawesi Indonesia; 10 Museum Für Naturkunde - Leibniz Institute for Evolution and Biodiversity Science, Invalidenstraße 43, 10115 Berlin, Germany; 11 University of Papua New Guinea, Port Moresby, Papua New Guinea; 12 Department of Entomology, SNSB‐Zoologische Staatssammlung München, Münchhausenstrasse 21, 81247 Munich, Germany

**Keywords:** Beetle evolution, Dytiscidae paleogeography, Island biogeography, Melanesia, Foja Gauttier Mountains, Ultramafic rocks, Water beetle phylogenetics

## Abstract

**Background:**

The New Guinean archipelago has been shaped by millions of years of plate tectonic activity combined with long-term fluctuations in climate and sea level. These processes combined with New Guinea’s location at the tectonic junction between the Australian and Pacific plates are inherently linked to the evolution of its rich endemic biota. With the advent of molecular phylogenetics and an increasing amount of geological data, the field of New Guinean biogeography begins to be reinvigorated.

**Results:**

We inferred a comprehensive dated molecular phylogeny of endemic diving beetles to test historical hypotheses pertaining to the evolution of the New Guinean biota. We used geospatial analysis techniques to compare our phylogenetic results with a newly developed geological terrane map of New Guinea as well as the altitudinal and geographic range of species (https://arcg.is/189zmz). Our divergence time estimations indicate a crown age (early diversification) for New Guinea *Exocelina* beetles in the mid-Miocene *ca.* 17 Ma, when the New Guinean orogeny was at an early stage. Geographic and geological ancestral state reconstructions suggest an origin of *Exocelina* ancestors on the eastern part of the New Guinean central range on basement rocks (with a shared affinity with the Australian Plate). Our results do not support the hypothesis of ancestors migrating to the northern margin of the Australian Plate from Pacific terranes that incrementally accreted to New Guinea over time. However, our analyses support to some extent a scenario in which *Exocelina* ancestors would have been able to colonize back and forth between the amalgamated Australian and Pacific terranes from the Miocene onwards. Our reconstructions also do not support an origin on ultramafic or ophiolite rocks that have been colonized much later in the evolution of the radiation. Macroevolutionary analyses do not support the hypothesis of heterogeneous diversification rates throughout the evolution of this radiation, suggesting instead a continuous slowdown in speciation.

**Conclusions:**

Overall, our geospatial analysis approach to investigate the links between the location and evolution of New Guinea’s biota with the underlying geology sheds a new light on the patterns and processes of lineage diversification in this exceedingly diverse region of the planet.

**Supplementary Information:**

The online version contains supplementary material available at 10.1186/s12862-021-01764-2.

## Background

**Fig. 1:**
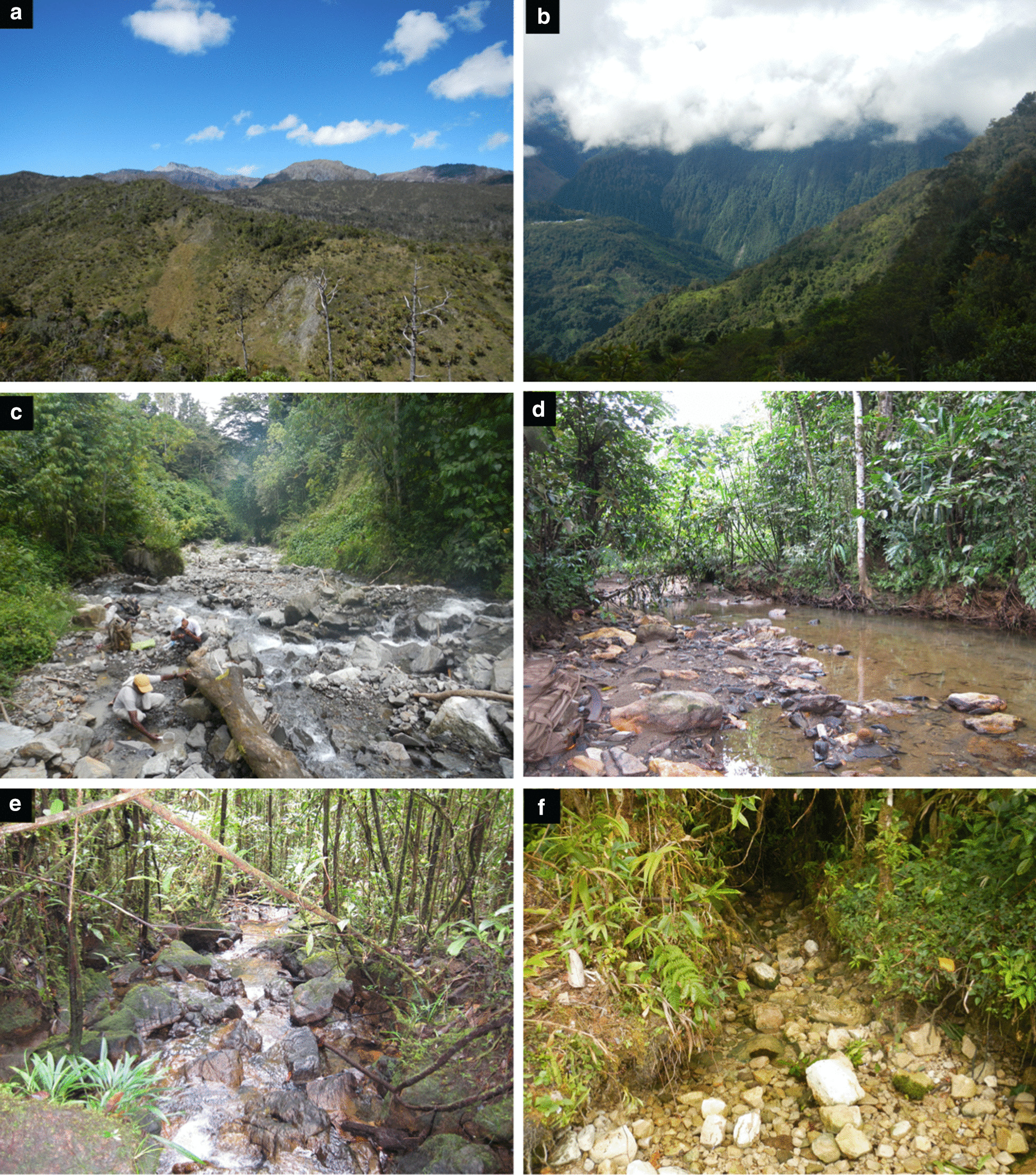
Snapshot of landforms and habitat diversity of New Guinea. **a** High summits of the Central Range including Mount Trikora (Wilhelmina); **b** upper montane forest on the southern slopes of the Central Range below the summit region of Mount Mandala (Juliana); **c** montane forest at Syoubrig, Bird’s Head, uplifted Australian Plate affinity rocks, 1,400 m; **d** lowland forest stream on Papuan Ophiolite Belt, south of Nabire, 340 m; **e** foothill forest stream on Papuan Ophiolite Belt, south of Nabire, 770 m; **f** creek in upper montane forest of Central Range, uplifted Australian Plate affinity rocks (Ok Sibil area). All photographs by M. Balke.

**Fig. 2:**
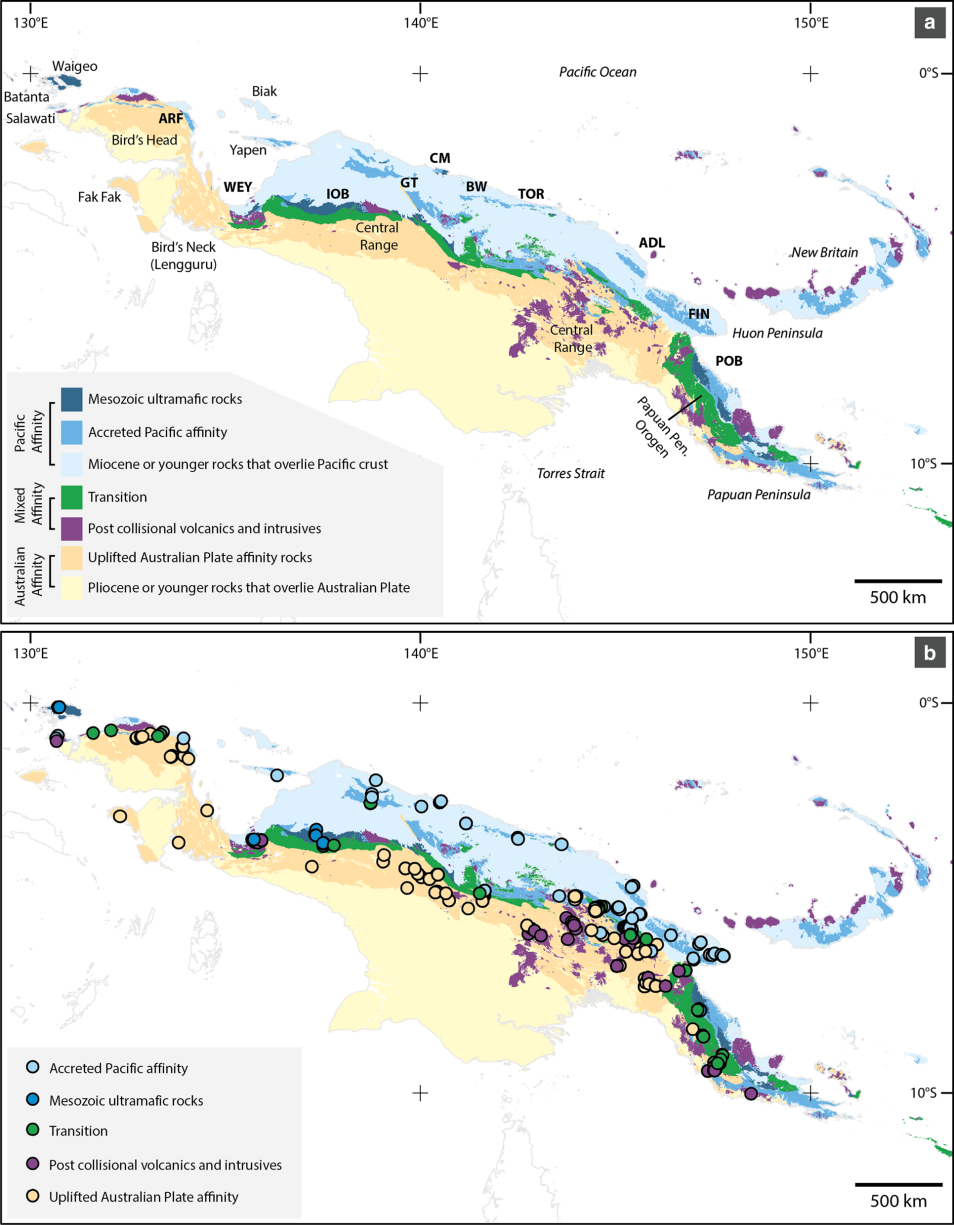
Geography of New Guinea and sampling localities. Custom build map using ArcGIS v10.4 and a comprehensive collection of geological maps described in the Methods section. The principal geological features are color-coded, see legend of Fig. 2 and Additional file 1 for further details. ARF (Arfak Mts), WEY (Weyland Mts), GT (Gauttier Terrane or Foja Mts), CM (Cyclops Mts), BW (Bewani Mts), TOR (Toricelli Mts), ADL (Adelbert Mts), FIN (Finisterre Mts), IOB (Irian Ophiolite Belt), POB (Papuan Ophiolite Belt). Sampling localities of the *Exocelina* individuals, dots colored according to the underlying geological terrane (lower panel)

New Guinea is the second largest island on Earth, featuring an exceedingly species rich biota, diverse climate zones and landforms [1], as well as a highly complex geological history [2, 3] (Fig. 1). Yet, what we recognize as New Guinea with its present extent of 800,000 km^2^ and mountains as high as 4800 m, only came about quite recently, most likely during the last few million years (Ma) of the planet’s history (e.g., [4, 5]). New Guinea would have looked much different in the past due to a combination of fluctuating global sea levels and various phases of tectonism which drove mountain building in some regions and subsidence in others [6]. For example, most of western New Guinea was submerged during the late Miocene–early Pliocene (i.e., ~ 7–5 million years ago, Ma) [7]. These changing landscapes are primarily due to the fact that New Guinea marks the northern margin of the Australian Plate, and that it also marked the northern margin of Gondwana for hundreds of millions of years, prior to its break-up (e.g., [2, 3, 6, 8–10]). This zone of interaction between tectonic plates implies that pieces of Earth’s crust have been progressively added to the island, as well as being torn from the island and laterally transported from west to east along large-scale faults over a long period of time (e.g., [6, 11–15]). This history is reflected in the rocks found on New Guinea today. Regions that share a similar age, history and affinity can be mapped and classified as a “terrane” (Fig. 2).

Increasingly detailed paleogeographic reconstructions and the availability of larger scale molecular phylogenetic analyses have created an opportunity to empirically investigate biodiversity patterns and processes, and possibly relate them to geological change. For instance, [7] used fossilized benthic foraminifera assemblages and geological data to reconstruct relative sea-level changes of the western New Guinea area (including the Bird’s Head Peninsula, Fig. 2). They show how paleodepositional environments changed through time. While the inference of areas above sea-level and regions of poor data coverage remains tentative, their evidence suggests a strongly varying configuration of land and sea in the region since the Carboniferous, with possible submergence of the entire region from the middle Miocene until a period of rapid uplift in the late Pliocene–Pleistocene. This work, together with other geological studies, indicates that the uplift and formation of the present-day, rugged, west New Guinea landscape is due to recent tectonism associated with the interaction between the Australian and Pacific plates, primarily during the past 5 Ma (e.g., [4, 13, 16–20]). These paleogeographic models have found an unexpected echo in recent empirical evolutionary studies. For instance, a population genomic study of a diving beetle species with a wide range across the Bird’s Head suggests that the strong geographic structure found could possibly be related to the area’s fairly recent fluctuations in land/sea configuration [21].

A series of recent papers on different groups of animals (e.g., [13, 21–40]; summaries in [33] and [41]) and plants (see e.g., [42] and references therein) identifies three major processes fueling biological diversification in New Guinea: (1) ancient diversification events on smaller proto Papuan islands including a proto Papuan Peninsula, or on the northern part of the Australian craton (see [27, 33, 43–45]), (2) more recent but substantial lineage diversification connected to the New Guinea orogeny (e.g., [41, 46]), or (3) formation of land in the north and south of the central highlands by various processes (volcanism, accretion/uplift of island arcs and ophiolites). The latter includes complex phylogeographic processes across the New Guinea lowland rainforest belts that led to the formation of allopatric species pairs (e.g., crowned pigeons: [47]) and orogeny related vicariance [48] (see Table 4).

In a series of innovative papers, biogeographers sought to directly link distribution patterns to such geological events, strongly motivating increased exchange between biologists and geologists over the past two decades (e.g., [39, 41, 49–52]). This is certainly the case to a large extent, but many clade diversification events might also result from biotic exchange across a more or less existing landmass, although composite and gaining altitude, as suggested by few recent analyses of insects [26, 41], frogs [32] and birds [25, 29, 47]. In that case, and related to the above process (3), the extant geography of the island might be structuring clades of species more than the geological formation on which they occur (see [41]).

In the broadest geological sense, New Guinea can be broken down into three terranes (Fig. 2).

1. Most of the northern coastline (“northern belt”) consists of rocks that were once part of the Pacific Plate and were pushed southward, onto the New Guinea margin (e.g., [6, 11, 12]) (“Accreted Pacific” and “Mesozoic ultramafic rocks” in our analyses, Table 1). The “Accreted Pacific” terranes contain segments of volcanic arcs that were part of the Pacific Plate during the Eocene–Miocene. The “Mesozoic ultramafic rocks” represent areas where sections of Cretaceous and older seafloor have been uplifted and pushed onto northern New Guinea (i.e., ophiolites) (e.g., [3, 14, 53, 54]).
2. Much of the Bird’s Head Peninsula, the Central Range, parts of the Owen Stanley Range (Papuan Peninsula), and the region to the south of this mountain belt all comprise rocks that were once part of Gondwana with many being compositionally and age equivalent to rocks found in northern Australia (e.g., [8, 9, 55]) (“Uplifted Australian Plate affinity rocks” in our analyses, Table 1).
3. Between these two zones is a ‘transition zone’ (or transition belt) where there is a mixture of deformed rocks of Pacific and Australian plate affinities that have been tectonically juxtaposed [56, 57] (“Transition” in our analyses). There are also small areas of volcanic and intrusive rocks that erupted or were emplaced after plate collision (“Post collisional volcanics and intrusives” in our analyses). These igneous rocks are relatively young and typically occur as small regions within the other terranes (Table 1). Readers should note that the “Post collisional volcanics and intrusive rocks” are not related to the arc volcanic/accreted Pacific material.

**Table 1:** Explanation of the scheme that was used to classify particular regions of the geological map to a particular terrane

Geological terrane	Description
Uplifted Australian Plate affinity	Rocks that are Miocene or older and have always been or were once part of the Australian Plate
Transitional	Metamorphic rocks—typically of Cretaceous age. Note however, these typically have poor age control and more often than not were classified on the basis that these are deformed/metamorphic rock
Mesozoic ultramafic rocks	Refers to Cretaceous or older ultramafic rocks
Accreted Pacific Plate affinity	Cretaceous to Early Miocene mafic volcanic rocks that likely accreted to New Guinea's northern margin during the Cretaceous, or during the Eocene–Oligocene. This classification contains some ultramafic rock
Post collisional volcanics and intrusives	Refers to volcanic and plutonic rocks that are Miocene age or younger
Miocene or younger rocks that overlie Pacific Plate	Refers to sedimentary rocks and carbonates that are Miocene or younger that overlie what was once likely part of the Pacific Plate (and therefore assumed to also be representative of the ‘Accreted Pacific Plate affinity’ category)
Pliocene or younger rocks that overlie the Australian Plate	Refers to sedimentary rocks and carbonates that are Pliocene or younger that overlie what is or was once part of the Australian Plate (and therefore assumed to also be representative of the ‘Uplifted Australian Plate affinity’ category)

The juxtaposition of ancient Australian Plate rocks with younger volcanic arcs and sections of ocean floor provide a first-order control on New Guinea’s unique biogeography [28, 33, 41, 42, 50, 51].

Here, we aim at reviewing the idea of geology-driven lineage diversification, using a newly assembled terrane framework that outlines the distribution of the major geological features of New Guinea according to the current state of geological knowledge (Fig. 1). The material and methods section provides a detailed account on the geological and biogeographic background.

Three major historical hypotheses can be formulated. *H1* is diversification on ancient islands arcs that make up the present day north coast ranges since the mid-Miocene or earlier [33, 43], and related to that *H1A* that colonization of the little studied Foja Mountains occurred around that time. Hypothesis *H2* is that older clades should occur in, or nearby, areas of ophiolite and ultramafic rocks (e.g., Mesozoic ultramafic rocks, Fig. 2; see e.g. [58]).

With focus on the vast Central Range of New Guinea, our hypothesis *H3* suggests an early diversification, possibly in an initial setting of a chain of islands, and subsequent colonization of surrounding areas such as the Bird’s Head and the Papuan Peninsula. More specific tests could be made under the following assumptions: (hypothesis *H3A*) the 1300 km long Central Range initially consisted of several islands, this implies localized radiations in different present-day highland blocks; (hypothesis *H3B*) there is a temporal sequence from west to the east; (hypothesis *H3C*) mountains of eastern PNG, the Papuan Peninsula, have existed as a separate island before 25 Ma and do therefore harbor fauna older than expected based on the above geological scenarios, and served as source area for other parts of New Guinea.

Furthermore, we investigate the role of geography in structuring clades by asking if geographic structure of extant clades is caused by geological history or more recent processes.

In order to test these hypotheses, we use geospatial analysis techniques to compare the results of a comprehensive molecular phylogeny of New Guinea *Exocelina* diving beetles (Coleoptera, Dytiscidae, Copelatinae) with the location, altitude and underlying geology of where each beetle was sampled. These beetles have recently been developed as a fruitful study system to investigate fine to large-scale patterns of evolution across New Guinea and also Melanesia [21, 41, 46, 59, 60]. Greatly benefiting from repeated collecting campaigns combined with fast integrative taxonomy, knowledge of this genus has steadily increased over the past few years. To date, 152 species have been described from New Guinea and its satellite islands (e.g., [61, 62]), 151 of which form a monophyletic group (see [41, 46]. The majority of species outside New Guinea occur in Australia and New Caledonia, with single species e.g., in Hawaii, China, Peninsula Malaysia [63] and Vanuatu. Most species are found in a variety of running water associated habitats (but avoiding water current), only a few Australian and only one New Guinea and New Caledonian species, respectively, inhabit pools or swamps [46]. For the New Guinea radiation, typical habitats include small stagnant water bodies on riverbanks, the interstitial, water filled rock holes in stream beds, or the tiniest wet spots beside creeks, or even above what most people perceive as the actual spring. The beetles are carnivorous, and as far as known, prey on virtually anything they can, including pieces of fish that were placed as a bait (M. Balke, unpublished). Feeding preferences remain unknown. Larvae have not been found to date, suggesting a rather hidden or unusual way of life. *Exocelina* diving beetles would preferably hide in gravel, among pebbles or underneath leaves (Fig. 1). In New Guinea, they occur from close to sea level up to 3000 m altitude (https://arcg.is/189zmz). The beetles are capable of flight, yet most species have small ranges. A few are comparably widespread, according to species delineated based on morphological characters (e.g., [64]. Phylogenomic data did nevertheless reveal extreme levels of very recent geographic population differentiation in one supposedly widespread species [21]. Such differentiation occurred between sites as close as 40 km, with no obvious major landscape obstacles. While the beetles can fly, it remains to be tested empirically which flight pattern they show and how far they fly—flight might for example only occur along a particular creek or river bed until the next suitable spot was reached. Lam et al. [21] suggested that dispersal might be rare, and possibly limited by barriers such as marine intrusions, but again, this remains to be tested.

However, being lotic taxa with the above properties known, as well as being easily detected in the field, suggested *Exocelina* as a feasible group to study across a vast area.

## Results

### Geospatial data

All our data, the terrane map and the regional classification are summarized in an online platform at https://arcg.is/189zmz.

### Phylogenetic inference and node age estimation (Fig. 3)

**Fig. 3:**
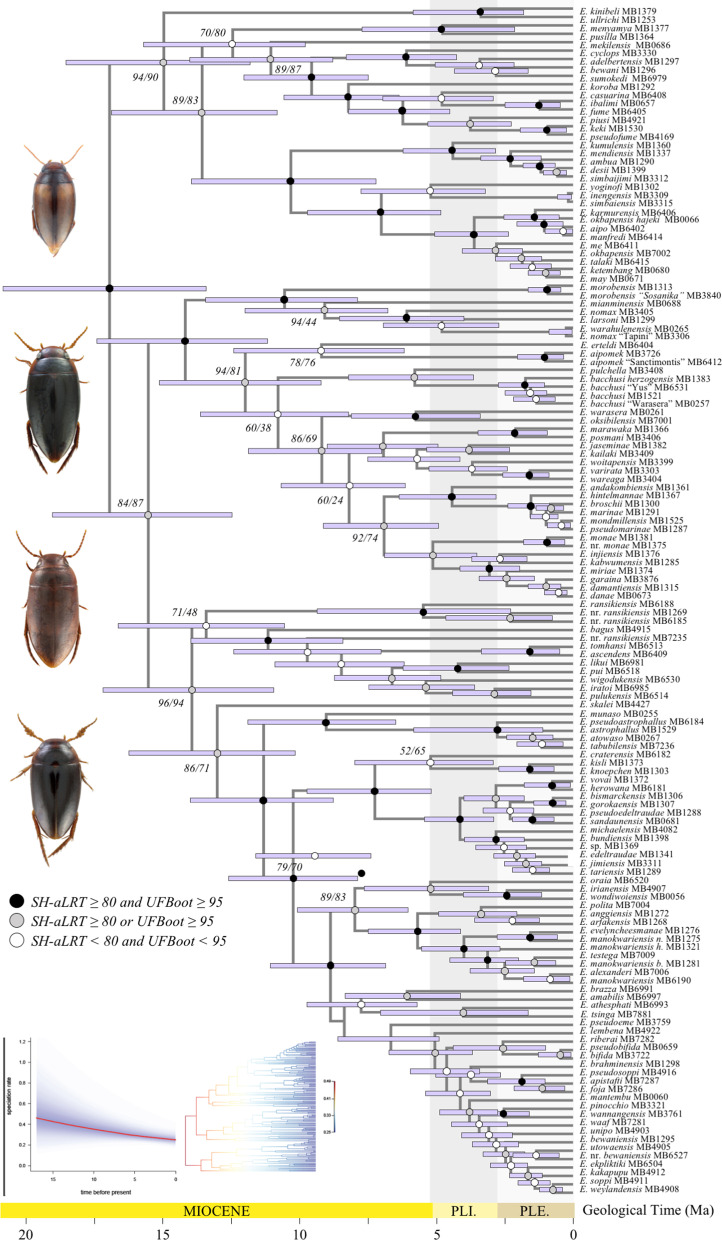
Bayesian divergence time estimates of *Exocelina* diving beetles. Chronogram presenting median ages across New Guinean *Exocelina* derived from the best BEAST analyses based on MLE comparisons (12 uncorrelated lognormal relaxed clocks with a birth–death tree model). 95% credibility intervals are given in purple horizontal rectangles. Branch support derived from the best IQ-TREE ML tree search is given (SH-aLRT/UFBoot). A summary of the BAMM analyses is inserted in the bottom left corner of the figure showing a decrease in speciation rate through time.

The results of the best scoring IQ-TREE ML search are presented in detail in Additional file 1: Appendices 5, 6. The topology for the monophyletic New Guinean radiation (SH-aLRT = 100/UFBoot = 100) is presented in Fig. 3 along with the results of the best BEAST analysis (Table 2). All dating analyses conducted in BEAST converged well as indicated by high ESS values (> 200) for all estimated parameters. The comparison of MLE suggests that the analysis including 12 clocks and a birth–death model is the best fit (Table 2), and therefore we present the results of this analysis in Fig. 3 (see also Additional file 1: Appendices 7, 8). The crown of Copelatinae is dated back to *ca.* 92 Ma (95% credibility interval 76–110 Ma). The split between the crown of *Exocelina* (46 Ma, 95% CI 37–56 Ma) and the crown of *Liopterus* and *Capelatus* (67 Ma, 95% CI 46–88 Ma) is dated back to *ca.* 77 Ma (95% CI 59–98 Ma). The split between the New Guinean radiation (17 Ma, 95% CI 14–21 Ma) and its sister clade of Australian subterranean species (24 Ma, 95% CI 16–32 Ma) is dated back to *ca.* 32 Ma (95% CI 25–39 Ma).

**Table 2:** Comparison of BEAST analyses using marginal likelihood estimates

Analysis	Clocks	Tree model	SS MLE	PS MLE	Crown Copelatinae	Crown *Exocelina*	Crown NG *Exocelina*
A1	2	Yule	− 68127.998	− 68127.821	100.16 (81.05–116.80)	49.31 (38.41–60.43)	20.57 (14.28–23.07)
A2	2	Birth death	− 68096.052	− 68096.531	92.84 (74.25–111.88)	39.16 (29.93–49.40)	14.78 (11.15–18.79)
A3	12	Yule	− 67442.242	− 67437.208	99.39 (82.36–116.47)	53.88 (43.43–63.25)	20.97 (16.84–25.22)
A4	12	Birth death	− 67417.937	− 67414.115	92.63 (75.59–110.38)	46.00 (37.19–55.83)	17.06 (13.53–20.98)

### Patterns identified using geospatial analytical technique

Each value within the sample database (Additional file 1: Appendix 2) was classified into one of five groups according to the output of the ArcGIS Grouping Analysis tool (Additional file 1: Appendix 9). These results have been drawn as a series of maps (Additional file 1: Appendix 9) and were plotted as a series of 2D histogram “heat maps” (Additional file 1: Appendix 10). Group 1 consists of samples collected from regions that are < 500 m altitude and dominantly Accreted Pacific Plate affinity. Group 2 broadly consists of values that reside on Accreted Pacific Plate affinity rocks, and altitudes between 500 m and 3500 m. Group 3 broadly consists of sites that reside on dominantly Transitional zone rocks (as well as some post-collisional igneous rocks) at altitudes of 0 m to 1500 m. Group 4 consists of samples collected from regions of dominantly Australian Plate affinity, as well as at altitudes of < 1500 m. Group 5 consists of sample sites > 1500 m, and from a mixture of rock types (mostly Accreted Australian Plate affinity as well as ultramafics and post collisional igneous rocks).

### Ancestral state reconstructions

**Fig. 4:**
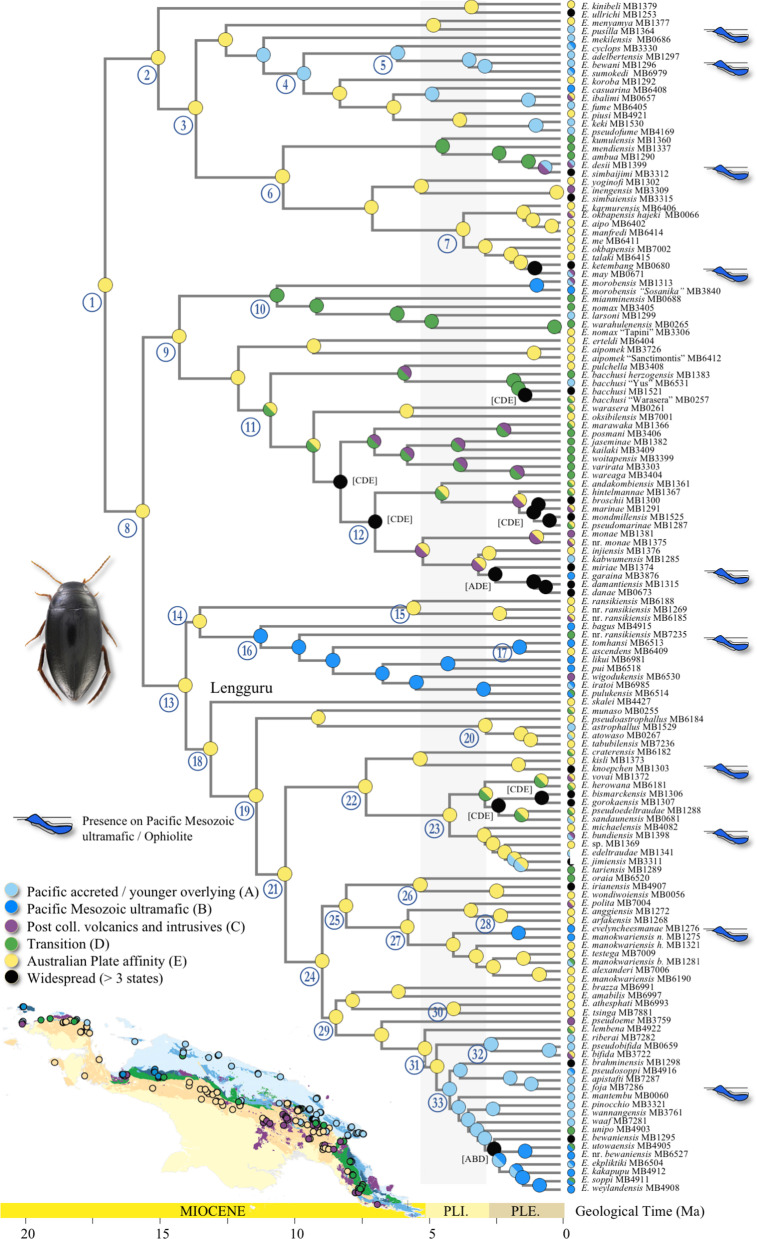
Maximum likelihood ancestral state estimation for geology coding

**Fig. 5:**
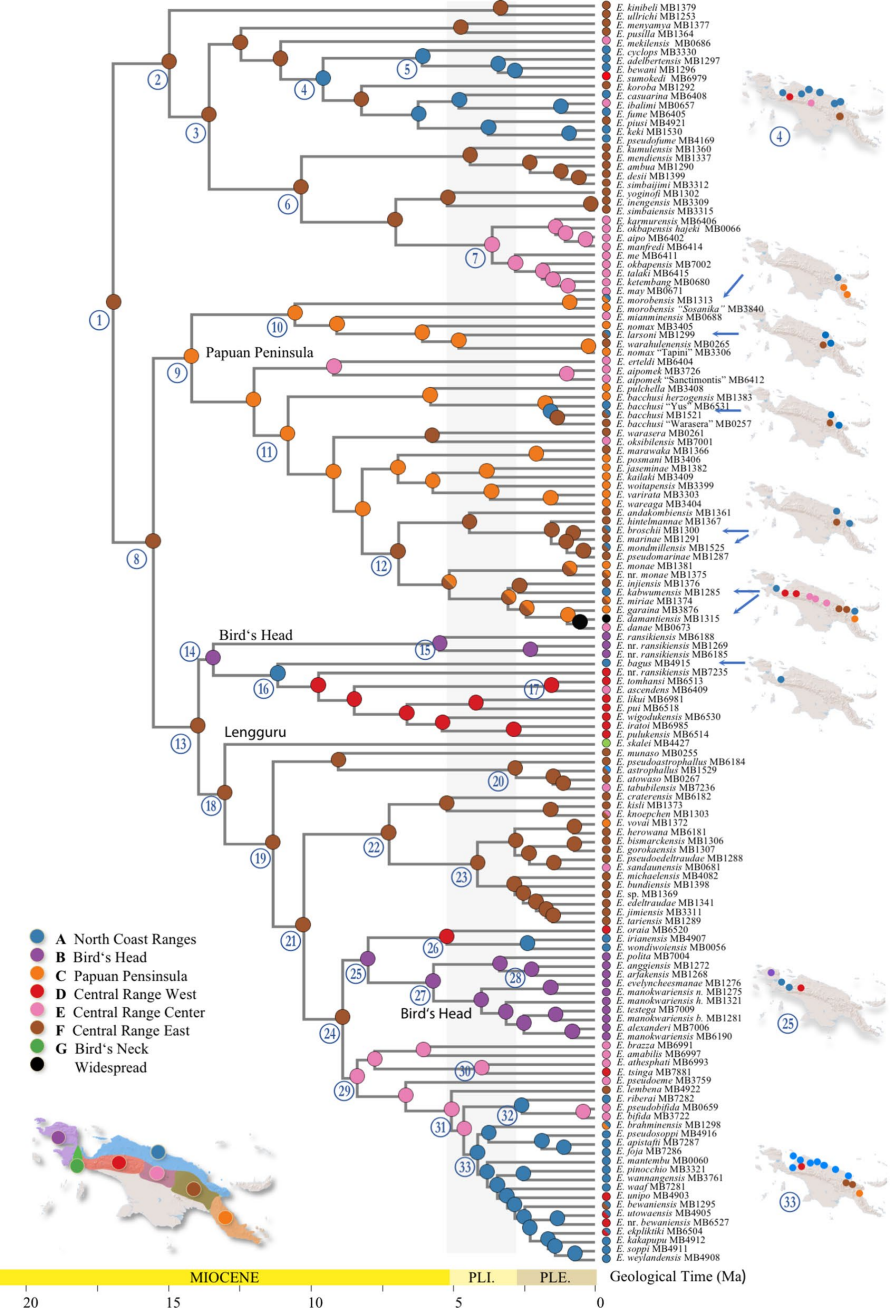
Maximum likelihood ancestral state estimation for geography coding. Inlay map shows the regions coded for the analysis, numbered nodes are discussed in the text; the maps to the right of the terminals visualize the distribution of a single species (one arrow) or a clade of species (two arrows or node number), the dot color corresponds to color code of geographic area.

The output files from the BMM analyses are provided in the supplement, including additional trees detailing the node distributions (Additional file 1: Appendix 11). For clarity, Figs. 4 and 5 only show the most likely states (MLS) at the nodes.

### Geology (Fig. 4)

**Fig. 6:**
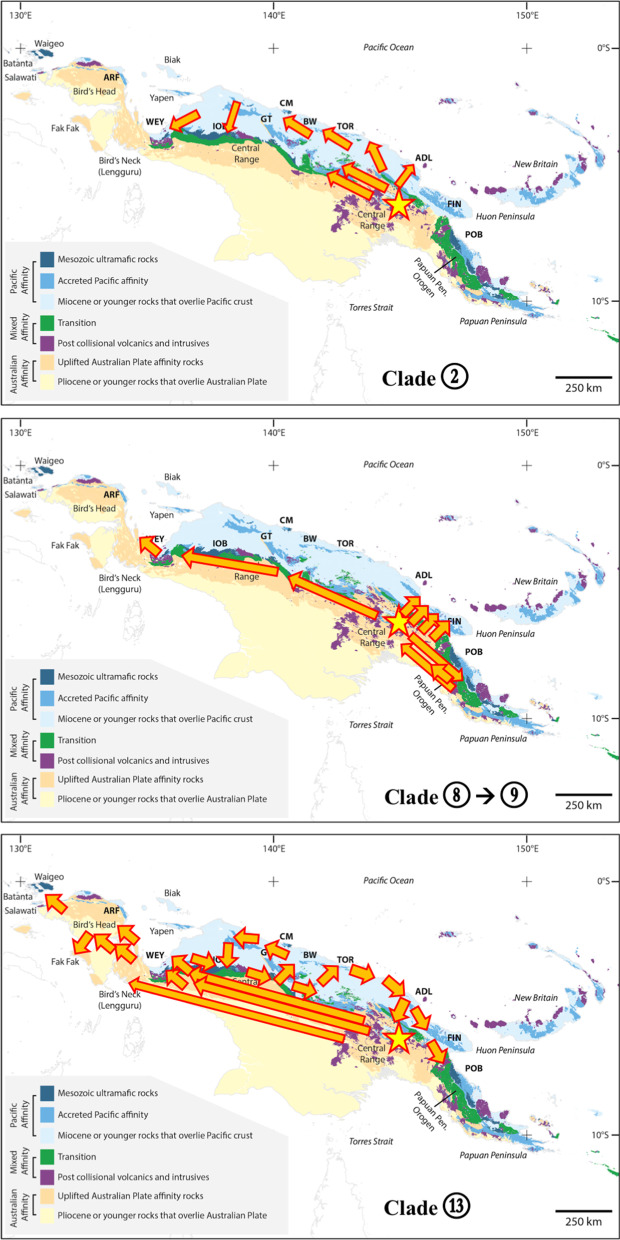
Graphical summary of major biogeographic processes inferred in the present paper. Note, the length and position of arrows indicates simplified processes, length and direction generalized

From the first node of the tree (node 1) and along its entire backbone (e.g., nodes 8, 13, 18, 19, 21, 24, 29), we almost unambiguously infer presence on geological formations with Uplifted Australian Plate affinity as the ancestral state (MLS node 1: E, prob. 0.984; others CE, 0.003, AE 0.009). This translates to occurrence on or along the orogenes of the Bird’s Head, the Central Ranges as well as the Papuan Peninsula (see also Fig. 6, geography). From *ca.* 10 Ma, we find clear evidence for some degree of species level diversification in the Transition areas (“coded as D) along the northern margins of the mountains as well as the interwoven post collisional volcanics and intrusives (“C”), e.g., at node 10 (MLS: D, prob. 0.291; followed by C, prob. 0.259). At approximately the same time, in a clade containing species from the Papuan Peninsula, the north coast ranges as well as the Central Range, we find increased interactions with these areas C and D (node 11, MLS: CE, prob. 0.207; E, prob. 0.222; C, prob. 0.148), followed by a number of nodes with ambiguous states but including C and or D (at node 7, this was estimated at *ca.* 5 Ma (MLS: C, prob. 0.758).

Interactions with the Pacific accreted/younger overlying rocks (area A), might have occurred early on, with some ambiguity, from *ca.* 12–10 Ma (node 4), less ambiguously so from *ca.* 6 Ma with a clade with species from the Cyclops, Adelbert and Bewani Mts as well as the foothills of the Central Range West (node 5). In that clade, we also found two species on the Pacific Mesozoic ultramafics (Ophiolite), one in the Cyclops Mts and one in the foothills of the Central Range West. Species on the Pacific Mesozoic ultramafics also occur more towards the tip nodes across the tree (see Fig. 4, obduction pictograms above tree terminals). At *ca.* 11 Ma, at node 16, we find the oldest shift from Australian Plate to the Pacific Plate/Mesozoic ultramafics (MLS: B, prob. 0.829; E prob. 0.786; BE prob. 0.725), these are species from the Irian Ophiolite Belt (IOB) as well as the species *E. bagus* in Mt. Gamey area in the foothills north of the Weyland Mts. *Exocelina bagus* is the sister to rest of the clade at node 16 (thus dated at 11 Ma). The wider Mt. Gamey area (north of the Weyland Mts) also has a number of species in the clade above node 33 assigned to B, ultramafics, but with more recent presence there, starting < 3 Ma.

The geological distribution of each taxon is also visualized in a 2D histogram “heat map” and plotted onto the phylogenetic tree in Additional file 1: Appendix 10.

### Geography (Fig. 5)

The MLS at the first node (node 1) was F, occurrence in the Central Range East (probability 0.838), followed by C, Papuan Peninsula (0.053). The clade at node 2 also had MLS F (0.991, or EF, 0.004). In that clade, starting at *ca.* 15 Ma, species diversified in the eastern bloc of the Central Range in PNG, and from there from *ca.* 12–10 Ma in the north coast ranges (area A, e.g., node 4). From *ca.* 3 Ma we infer exchange from the north coast ranges towards the western part of the Central Range (clade 5). There is repeated interaction with the central part of the Central Range (E, Star Mountains), which from *ca.* 4 Ma also show a local radiation (in the clade above node 6).

The ancestral state reconstruction for the next major node (node 8) suggests the MLS being F (0.656), followed by C (0.139), suggesting occurrence in either the Central Range East, or Papuan Peninsula. The clade at node 9 (MLS C), containing most of the species from the Papuan Peninsula, is comparably dynamic in terms of geographic distribution, with interactions with areas A, D, E and F. The rather unambiguous diversification in the Papuan Peninsula was inferred from *ca.* 14 Ma. From there, we find interaction with neighboring north coast ranges (e.g., Huon, Adelbert and S of Madang), and the Central Range from the east towards the west into Indonesian Papua. One recent species, *E. damantiensis* (< 3 Ma) is widespread across the island (ACDEF) (in clade 12).

Moving up the tree backbone, nodes 13, 18, 19, 21 and 24 have the same MLS: F, Central Range East. Major transitions occur *ca.* 13 Ma towards Bird’s Head and Central Range West (nodes 14, 16), and the Bird’s Neck (Lengguru, at node 18). The single species sampled from the Bird’s Neck (Lengguru) region, *E. skalei*, is therefore rather isolated and separated from its species rich sister clade *ca.* 13 Ma (node 18). Later, at *ca.* 9 Ma, there are transitions to E, Central Range Center (node 29), and the Bird’s Head and adjacent areas (node 25) (see below).

From node 13, diversification events appear well structured geographically. The clade at node 14 has a subclade with three Bird’s Head species, and one clade (node 16) mainly with species from the Central Range West (MLS A or D). This clade contains one species from neighboring north coast ranges (A, *Exocelina bagus*) and one from Central Range East (E, *Exocelina ascendens*, which is similar genetically and morphologically to *E. tomhansi* in area D) (node 17). At node 18, branches off one species from the Bird’s Neck or Lengguru extension of the Bird’s Head, sister to a clade at node 19 with MLS F (prob. 0.907), with local species diversification locally structured according to areas A, B, E and F. The clade at node 25 (MLS: B, prob. 0.623) contains species from the Bird’s Head region as well as three species coded A or D, however, from localities in close proximity to region “B”. The clade at node 29 (MLS: E, prob. 0.946) has species mainly in the Central Range Center (few in East or West). From there, at node 33 (MLS: A, prob. 0.858), we infer a range expansion into the north coast ranges, at the end of the Miocene, with species along the north coast including in Indonesia: Yapen Island, Weyland Mts foothills south of Nabire, Van Rees Mts, the Foja Mountains, and in PNG the Bewani, Toricelli, Adelbert and Herzog Mts. That range expansion was roughly out of Central Range east towards the north coast and then occurred in a roughly westward direction up to Yapen Island and the area south of opposite Nabire, which is especially species rich with at least 10 species originating in the past *ca.* 5 Ma.

Actual diversification in the Bird’s Head region and satellite islands Waigeo, Batanta and Salawatti began between *ca.* 9–6 Ma (nodes 15, 25), possibly older *ca.* 14 Ma (if species at node nodes 14 already occurred in the Bird’s Head region).

ed.

### Diversification rate dynamics estimation

The different BAMM analyses performed on the chronogram corresponding to the New Guinean radiation yields identical results. We recovered a scenario with no rate heterogeneity among New Guinean clades. Regardless of the number of expected shifts, the analysis of parameter posterior distributions recovered a single most credible shift configuration with no rate shift throughout the evolution of the New Guinean radiation. The phylorate summary analyses and rate through time plots suggested a continuously declining speciation rate through time. We present the results of the analysis with an expected number of shifts of 0.1 in Additional file 1: Appendix 13.

The RPANDA analyses recovered a model with both speciation and extinction rates varying linearly with time (BTimeVarDTimeVar_LIN, LnL = − 352,770) as the best-fit model for the evolution of New Guinean *Exocelina*. This model was a significantly better fit than other time-dependent and diversity-dependent models. The latter all indicated that the New Guinean *Exocelina* radiation is still far from its potential carrying-capacity (parameter K in Table 3). The preferred model recovered a slowdown of both speciation and extinction rates through time (Table 3).

**Table 3:** Results the diversification analyses conducted on the New Guinean *Exocelina* radiation

Model	Param,	Log Likelihood	AICc	ΔAIC	λ	α	μ	β	K
BCST	1	− 358,847	719,723	5,892	0,2405	–	–	–	–
BCSTDCST	2	− 358,847	721,780	7,949	0,2405	–	0	–	–
BTimeVar_EXPO	2	− 358,584	721,254	7,423	0,2241	0,01626	–	–	–
BTimeVarDCST_EXPO	3	− 358,584	723,342	9,511	0,224	0,01629	0	–	–
BCSTDTimeVar_EXPO	3	− 358,847	723,868	10,037	0,2405	–	0	0,012	–
BTimeVarDTimeVar_EXPO	4	− 358,584	725,460	11,629	0,2241	0,01628	0	0,03216	–
BTimeVar_LIN	2	− 358,634	721,355	7,524	0,2236	0,00395	NA	–	–
BTimeVarDCST_LIN	3	− 358,634	723,442	9,611	0,2235	0,00396	0	–	–
BCSTDTimeVar_LIN	3	− 358,891	723,955	10,124	0,2402	–	0	0	–
BTimeVarDTimeVar_LIN	4	− 352,770	713,831	–	0,0138	0,46044	0,35132	0,40155	–
DDL	2	− 358.622	721.331	7.5	0.273	–	–	–	786.86
DDL + E	3	− 358.526	723.226	9,395	0.3614	–	0.07543	–	375.61
DDX + E	3	− 358.578	723.329	9,498	0.5011	–	0.0474	–	Inf
DDL + EL	4	− 358.532	725.355	11,524	0.3391	–	0.05839	–	426.98

*Param* number of parameters in each model, Δ*AICc* the difference of AICc between any model and the best scoring model (i.e., BTimeVarDTimeVar_LIN), *λ* speciation rate at present, *α* dependency of speciation rate on time time (positive value indicates a slowdown of speciation rate, negative value indicates an acceleration of speciation rate), *μ* extinction rate at present, *β* dependency of extinction rate on time (positive value indicates a slowdown of extinction rate, negative value indicated an acceleration of extinction rate), *K* carrying capacity (species richness) estimated for diversity-dependent models

## Discussion

### Phylogenetic inference, divergence times and diversification dynamics

Within *Exocelina*, our topology slightly differs from earlier studies based on overlapping but more reduced molecular datasets [41, 65], they are however largely consistent with the latest placement of the genus [46] in Copelatinae (see Additional file 1: Appendix 5). Our results recover three main clades (2, 9 and 13). The monophyly of these lineages and their relationships are strongly to moderately supported, representing substantial progress in the establishment of a robust evolutionary tree for the genus *Exocelina*. Several morphological species-groups (MSG) appear paraphyletic or polyphyletic suggesting the need for a revision of species-group composition and key synapomorphic characters. The largest MSG is the *E. ekari* group, here recovered at node 18.

In terms of divergence times, our estimates shed a new light on *Exocelina* evolution, with contrasting results from earlier studies. Here, we estimated the crown age of New Guinean Exocelina ca. 17 Ma, previous studies suggested ca. 5 Ma (3.74–7.07 Ma) [46] or 9 Ma (5.51–12.50 Ma) in [41].

These observed discrepancies are largely due to the methodology used to obtain absolute divergence times using molecular clock calibrations. Earlier studies mostly relied on beetle rates of evolution or fossil calibrations placed in the outgroups, while the current one relies on secondary calibrations applied across a large selection of lineages and derived from a robust fossil-based evolutionary framework of diving beetles [66]. Hence, we believe these new estimates to be much more in line with the evolutionary history of these diving beetles than earlier estimates. Based on these new estimates, we infer that the three main clades 2, 9 and 13 diverged in the mid-Miocene *ca.* 15 Ma. The rest of diversification was gradual up to the last million years within the Pleistocene.

This is confirmed by BAMM analyses suggesting a diversification regime with no rate shifts and a continuously declining speciation rate through time. Our RPANDA analyses across the entire New Guinean radiation confirm this result with the best-fit model suggesting declining speciation and extinction through time (Table 3). This diversification rate regime was already suggested by [46] who analyzed the role of island colonization on diversification rate regimes in *Exocelina*. The authors showed that a significant increase in diversification rates occurred as soon as (proto) New Guinea was colonized and that this early burst in diversification was followed by a slow but steady decrease in speciation rates through time. This pattern substantiated here with a more extensive taxon sampling and different methods invalidates the hypothesis of diversity being bound by the availability of suitable habitat as suggested in [46]. Our DDD analyses recover a carrying-capacity that is far greater than the current and expected species richness in *Exocelina*. As a result, the declining diversification rate in *Exocelina* appears to be disconnected from diversity-dependent processes. The diversification dynamics in New Guinean *Exocelina* follows a model with an early burst in diversification coinciding with the colonization of New Guinea emergent landmasses out of Australia, followed by a progressively declining diversification pace. Though many processes may be responsible, competition possibly drove body size segregation in at least one instance. On lower elevations, species of the closely related genus *Copelatus* commonly occupy the same microhabitat as *Exocelina*. With *Copelatus* species being larger than *Exocelina*, competition between the two in sympatry may limit the potential number of *Exocelina* species in such habitats [46]. However, this remains to be tested with additional data, and thus, our results need to be carefully interpreted in light of the current debate regarding the potential pitfalls when estimating diversification rate dynamics from extant phylogenies [67].

Diversification processes are lineage idiosyncratic per se. Another pattern is, for example, a slight increase of diversification rates possibly related to the colonization of newly emerging landmasses in the proto Papuan archipelago. This was suggested for microhylid frogs, with an inferred temporal sequence of rate increases *ca.* 17–15 Ma (“East Papuan Composite Terrane”), *ca.* 11–6 Ma (Bird’s Head region), *ca.* 2 Ma (north coast ranges) [45]. These authors suggest that the dating and its relation to geological processes must be taken with caution, as in any other study including ours. The biological patterns emerging from such analysis are nevertheless significant and beg for explanation.

### Biogeography—testing the new terrane interpretation

We used our geospatial model for New Guinea to make inferences of ancestral states and their evolution in time and space for species occurrence on geological terranes, elevational distribution and according to their recent geographic distribution. “Geology” is essentially addressing historical processes and assuming lineage diversification on land very different in configuration in terms of size and position (and altitude) than present-day New Guinea. “Geography” seeks to make inferences based on the presently observed distribution of species, also invoking geological history (because recent areas if inferred as ancient distribution implies presence of at least some land), but possibly also much more recent processes.

### Evolution in the north coast region

Our hypothesis H1 suggested early evolution on (volcanic) island arcs adrift in the Pacific, prior to collision of these areas of Pacific affinity with the northern Australian plate margin (e.g., the Solomons Arc, work on aquatic bugs, [51] see also work on cicadas [49, 68]). The geography inference reveals two clades showing more pronounced species diversification along the north coast ranges (around nodes 4/5, and 33). This diversification process might have begun as early as *ca.* 9 Ma, with more species originating from *ca.* 4 Ma. During that time, we find interactions between north coast ranges and usually nearby parts of the Central Range (west, center or east, Fig. 5). Species across the tree have repeatedly colonized the north coast ranges from different blocks of the Central Range or the Papuan Peninsula next to them. The oldest such split was inferred at node 13 between a species in the Weyland foothills and a group of species in nearby Central Range West. The geology inference recovers a similar picture. At node 33, *ca.* 5 Ma, we infer a switch from occurrence on Australian Plate affinity to Accreted Pacific Plate accreted affinity (and younger overlying sedimentary sequences). Within that clade, the uplifted Australian Plate areas as well as the Transition belt and Post collisional volcanics were more recently colonized. In the clade around node 4, we infer an earlier presence (*ca.* 11 Ma) on areas of Pacific affinity than by simply looking at the Geography coding (*ca.* 9 Ma), because geologically, some of the northern parts of the Central highlands are also rocks of Pacific affinity. In summary, we find geographic as well as geological structure that confirms an important role of areas of Pacific affinity for the diversification of *Exocelina* diving beetles. Under the assumption that present day New Guinea only formed in the past few million years, this might indeed have been in a setting of a proto Papuan archipelago, with smaller islands existing close enough to the northern margin of the Australian plate to allow repeated exchange of biota in both directions. This scenario appears realistic considering that *Exocelina* diving beetles have frequently been observed by us flying, and are abundantly caught in flight interception traps [61]. Hypothesis *H1* is thus partially supported, although the ancestral area is unambiguously uplifted Australian Plate rocks (in terms of geology) of the central range (in terms of geography). This also confirms the results of other biogeographic studies, in particular those assuming a biogeographic origin on the Australian craton and then colonization and diversification on smaller islands to the north [27, 28, 33–35, 43–45, 48].

The Foja Mountains of the Gauttier Terrane have never been studied in a comparative biogeographic framework. We find three colonization events, only in the past < 3 Ma in the clade at node 33, out of other areas of Pacific affinity of the north coast ranges. Note that parts of the Foja Mountains might feature underlying Transition zone material. In any case, Hypothesis *H1A*, the Foja Mountains as a museum of diversity whereby diversity would have been accumulating for millions of years, does not seem credible in our specific study group.

### The role of ultramafic/ophiolite rocks and the Papuan Arc scenario

Hypothesis *H2* suggests that some of the older clades should occur in, or nearby, areas of ophiolite and ultramafic rocks. These are the areas biogeographers sometimes refer to as the ancient, oceanic Papuan Arc (15–11 Ma) [39, 51, 58]. Geographically, the ultramafic/ophiolite rocks occur in all the major regions we have coded, but we do not infer a strong geographic signal. Rather, we find close affinity with species from rocks of Pacific Plate affinity, Australian Plate affinity, Transition or Post collisional volcanics. This agrees with the actual geological processes that saw continued tectonic activity along the plate boundary, which resulted in further uplift and the tectonic juxtaposition of different terranes against one another along major fault zones, particularly after *ca.* 6 Ma (Fig. 4). However, we also infer an older collision event (*ca.* 12 Ma), at node 16, with a transition from species on Uplifted Australian Plate rock onto ultramafic/ophiolite. Within that clade, we find geographic interaction between the Central Range West and East in a vicariant species pair (node 17). In terms of geology, some species within that clade are found on Uplifted Australian Plate, Transition or Post collisional volcanics, also predicted by the emplacement process. The geographic distribution of species in clade 16 includes the Central Range West + a nearby north coast range (North Weyland). This suggests the comparably early existence of some amount of land, possibly several islands, in that region.

Increased sampling of that particular region would most likely rather suggest Uplifted Australian Plate rocks as MLS at node 16 as we mainly sampled immediately north of the Australian Plate formations, close to the last northern ridge of the Central Range, and then a transect down the northern slopes. The entire actual highland region of Central Range West remains essentially unsampled. The single species from its southern slopes of Central Range West (node 30, *E. tsinga*), only *ca.* 60 km south from our northern localities, is a species vicariant with a southern slope species from further east (*E. athesphati*) close to the PNG border. In summary, we did not find support for Hypothesis *H2*. While there might have been diversification events involving ultramafic/ophiolite rocks, their very emplacement process closely relates them with the later collision, uplift and fault movement between the Pacific and Australian plate affinity rocks.

Ultramafic rocks do, however play an important role in generating mountain biodiversity [69], as their soils promote endemic and diverse plant radiations. Streams on ultramafic rocks could be referred to as naturally polluted with heavy metals, so that aquatic fauna might require species physiological adaptations. This was suggested for New Caledonian caddisflies which radiated in streams on ultramafic rocks [70], or for the New Caledonian flora where ultramafic rocks harbor a disproportionately high fraction of endemic vascular plants compared to other areas [71]. In New Guinea, due to the accretion history, the ultramafic rocks make up the northern slopes of the Central Range and the Papuan Peninsula orogen. Aquatic insects are thought to be more diverse there, compared to the southern slopes, which are predominantly composed of limestone [51]. These authors suggest that this could be due to relief and elevation, which is much higher and steeper towards the south, having profound effects on stream diversity, type and shape. Here, intensive surveys of aquatic biota and abiotic factors including water chemistry could help to better untangle factors shaping diversity patterns in the New Guinea orogen.

### Evolution in the Central Range, Bird’s Head and Papuan Peninsula

Our hypothesis *H3* suggested an early lineage diversification in the present Central Range, possibly in a “proto Papuan archipelago” setting. Specific assumptions were formulated as follows: (*H3A*) the 1300 km long Central Range initially consisted of several islands, this implies localized radiations in different present-day highland blocks; (*H3B*) there is a temporal sequence from west to the east; (*H3C*) mountains of eastern PNG have existed as a separate island before 25 Ma and harbor old biota.

The geological inference we made clearly shows that the ancestral species of all major clades evolved on uplifted Australian Plate affinity rocks (coded as E), as well as rocks directly associated with the collision of material of Pacific affinity with the Australian plate margin (Transition, ultramafics and Post collisional volcanics, B, C, D). In addition, the geographic inference shows geographic structure in most clades. For *Exocelina* diving beetles, we have clearly shown that the early evolution likely took place on uplifted Australian Plate and associated rocks in the eastern part of the present-day Papua New Guinea highlands and the Papuan peninsula including the Herzog Mountains, as predicted by some previous authors [23, 28, 33, 42, 51] (see also Table 4). This area (E and C in our coding) corresponds to the “Woodlark Plate including East Papuan Composite Terrane” of [42], but as explained above, their concept of a Woodlark Plate confused modern-day plate delineation and the long-term tectonic evolution of New Guinea.

**Table 4:** Summary of major biogeographic trends in different taxa and across New Guinea

Taxon	Timing of early diversification in New Guinea	Area of early diversification	Early interaction Central Range/north coast areas?	Timing of Bird's Head colonization/diversification	Ancient diversification on or along "island arcs" (Proto Papuan Archipelago)?	Central Range as barrier causing N-S vicariance
*Flora*						
Schefflera, Shee et al. [42]	Late Oligocene, ca. 26 Ma	"Woodlark", i.e. present day Eastern PNG Central Range/*Papuan Peninsula*, and some PNG north coast ranges	Possibly	Not clear, possibly from early Miocene, diversification from c. 5 Ma	Not clear	Late Oligocene, ca. 26 Ma
*Insects*						
Diving beetles, this study	Early Miocene, ca. 17 Ma	*Present day Eastern PNG Central Range*/Papuan Peninsula	Yes	Mid/late Miocene	Early interaction since late Miocene, ca. 11 Ma, diversification from 5 Ma	Possibly late Miocene, ca 8 Ma
Mayflies (Corrarolo et al. 2019)	Eocene, ca. 40 Ma	Ambiguous: north coast region or southern lowlands	Yes	Late Oligocene/mid Miocene	Not applicable	Not clear
Ants, Janda et al. [26]	Mid-late Miocene 13–5 Ma	Unclear	Not applicable	Not applicable	Not applicable	No
Damselflies, Kalkman et al. [28]	Not dated	*Present day Eastern PNG Central Range/Papuan Peninsula*	Possibly	Not clear	Possibly	Not clear
Ground beetles, Liebherr [35]	Not dated	? Emerging Central Range	Possibly	Not applicable	Possibly	Yes
Aquatic bugs, Polhemus & Polhemus [51]	Starting late Eocene–Oligocene based on geological evidence	Not applicable	Possibly	Early tertiary?	Yes	Not applicable
Microveliinae aquatic bugs, Polhemus & Polhemus [50]	Late Oligocene—early Miocene, since 25 Ma	*Papuan Peninsula and its satellite islands*	Not applicable	Not applicable	Not applicable	Not applicable
*Mammals*						
Tree kangaroos, Eldridge et al. [36]	Late Miocene, ca. 7 Ma	Australia	No	? Late Miocene	No	Maybe, Pleistocene, ca. 2 Ma
*Herpetofauna*						
Scincidae New Guinea skinks, Slavenko et al. [72]	Mid Miocene, from ca. 14.4 Ma	Central Range/Papuan Peninsula	Mid Miocene, from ca. 11.6 Ma	Not applicable	No	No
Agamidae forest dragons, Tallowin et al. [48]	Late Oligocene—early Miocene, since 23 Ma	Australian craton	Early-mid Miocene, from ca. 23–10 Ma	Mid Miocene, ca. 14 Ma	Yes	Possibly late Miocene-Pleistocene
Frogs, Rivera et al. [45]	Late Oligocene to early Miocene, ca. 25-20 Ma	N Australian plate margin/Central Range	Possibly	Possibly	Possibly	Possibly
Lizards, Oliver et al. [44]	Early Oligocene, ca. 30 Ma	*North coast ranges and Papuan Pensinsula*	Early Miocene, ca. 22 Ma	Late Miocene ca. 10 Ma	Yes	Not clear
*Cyrtodactylus* Lizards, Tallowin et al. [33]	Early Miocene, ca. 24 Ma	Southern New Guinea/north coast ranges/from mid Miocene *Papuan Peninsula*	From early Miocene ca. 22 Ma	Late Miocene ca. 10 Ma	Yes	Not clear
Green python, Natusch et al. [30]	Not applicable	Not applicable	not applicable	Not applicable	Not applicable	Yes, starting late Miocene ca. 6–3 Ma
Microhylid frog, Oliver et al. [31]	Not dated	*Papuan Peninsula and its satellite islands*	Possibly	Not applicable	No	Check
Microhylid frog, Oliver et al. [32]	Early Miocene, ca. 17 Ma	Central Range	Early-mid Miocene, from ca. 17–10 Ma	Not applicable	Not applicable	Yes, late Miocene ca. 10 Ma
Catbirds, Irestedt et al. [25]	Late Miocene, 10 Ma	Australia or proto-New Guinea	Not applicable	Late Miocene/Pleistocene	Not applicable	Yes, late Miocene, ca 6 Ma to Pliocene
Freshwater turtle, Georges et al. [24]	Early Miocene, ca. 19 Ma	? NG southern lowlands	Not applicable	Mid Miocene	Not applicable	Yes, early Miocene, ca 17 Ma
Freshwater turtle, Todd et al. [37]	Late Miocene, 10–7 Ma	? N Australia/? NG southern lowlands	Not applicable	Late Miocene	Not applicable	Yes, late Miocene, ca 6 Ma
*Birds*						
Corvoid birds, Jønsson et al. [27]	Late Eocene, ca. 40 Ma	Proto Papuan Archipelago	Not applicable	Not applicable	Yes, late Eocene	Not applicable
Passerine birds, Aggerbeck et al. [43]	Early Oligocene, ca. 32 Ma	Australia/New Guinea	Not clear	Not applicable	Yes	Not applicable
Fruit doves, Cibois et al. [38]	Early Oligocene, ca. 32 Ma	Not applicable	Not applicable	Not applicable	Not applicable	Not applicable
Lories and lorikeets, Schweizer et al. [40]	Late Miocene ca. 10 Ma	Proto Papuan Archipelago	Not applicable	Not applicable	Not applicable	Not applicable
Songbrids, Moyle et al. [29]	Mid Miocene, ca. 15 Ma	Australia	Not applicable	Not applicable	Not applicable	Not applicable
Crowned pigeons, Bruxaux et al. [47]	Late Miocene ca. 5 Ma, root goes back to early Miocene	Not applicable	No	Between late Miocene ca. 5 Ma and Pleistocene	No	Yes, between late Miocene ca. 5 Ma and Pleistocene
*Fish*						
Rainbow fish, Unmack et al. [34]	Early Oligocene, ca. 30 Ma	Not applicable	Not applicable	Early Oligocene, ca. 32 Ma	Yes	Not clear, north/south vicariance dated at 27.0 Ma

Hypothesis *H3C* assuming eastern PNG as an evolutionary cradle, is here supported and in line with other biogeographic work cited above (and see [33, 48]), but based on our new terrane interpretation, we cannot be certain of the notion that diversification there happened on a large island drifting in the Pacific. However, this does not mean that we cannot envision an archipelagic setting in the region in the wake of the processing forming the present island and before.

In our example, different parts of the Central Range have been colonized by *Exocelina* in separate clades and at various geological times, e.g., from Central Range East (F) westwards to Central Range Center (E) (e.g., clades 6, 29), or towards Central Range West (D) (clade 16), and towards the Bird’s Head (14, 25).

We find geographic structure in our diving beetle tree, dating back millions of years, that suggests diversification on separate islands that are now part of the New Guinea central orogen, which gives support to our Hypothesis *H3A*. This would confirm the idea of early evolution in a proto Papuan archipelago, and we have clearly identified its gondwanan geological origin. We, however, do not find a clear directionality along the Central Range. We thus can not corroborate Hypothesis *H3B*. Interestingly, we do not find colonization out of the Bird’s Head region back towards the east. Our results here further highlight the important role and complexity of the New Guinean orogeny, and the orogen as a source of endemic Papuan diversity [33, 41, 48, 72] (Table 4). Unambiguous diversification for the Bird’s Head taxa is comparably recent, the crown clades are both estimated at *ca.* 6 Ma, with species mostly associated with uplifted Australian Plate rocks, after *ca.* 4–3 Ma also Pacific, ultramafic, Post collisional volcanics and Transition, suggesting the emplacement, uplift and/or exposure of these geological regions during that time. Presence in the Bird’s Head region might however date back up to *ca.* 13 Ma if stem lineage representatives already occurred in that region (nodes 14, 25). Our estimate would be in line with data e.g., from microhylid frogs [45], but estimates in other studies range from late Oligocene to Pleistocene (Table 4).

However, according to [7], continuous terrestrial habitat in the Bird’s Head might only be available since the Pleistocene. Previously, the extent and position of land was highly dynamic, but the continuous existence of smaller islands cannot be ruled out (see also phylogenomic data presented by [21]).

The Central Range remains little explored, and a much denser sampling is needed to understand smaller scale patterns of lineage diversification, in particular to unravel more recent population genetic processes (but see [21]) and the timing and rate of uplift. Towards node 7, we infer one colonization event of the Star Mountains that from *ca.* 4 Ma, gave rise to nine (known) species [73]. Such localized radiations highlight the role of individual larger and strongly structured mountain blocks, either as islands or sky islands [74].

### Emergence of general patterns?

Geological evidence suggests that the present day New Guinea landmass (i.e., land exposed above sea level) came into existence relatively recently (e.g., [7]). However, more and more publications on New Guinea’s flora and fauna date back their early diversification to the early Miocene or even late Eocene (Table 4), possibly in an archipelagic setting involving the emerging Central Range. This does not necessarily need to invoke early lineage diversification on oceanic islands arcs (e.g., a volcanic arc), but there is ample evidence for early interactions between the Australian craton and/or Central Range and oceanic areas that nowadays form the north coast ranges. This relates to our hypotheses H1 and H1A, ancient diversification along oceanic island arcs, for which our, and other studies (Table 4) deliver some support, but also highlight the role of dispersal between different areas.

The concept of ancient lineage diversification on an island arc of Mesozoic ultramafic rocks (our hypotheses H2) is too simplified; different areas of the Central Range harbouring older fauna might rather represent previously separate insular entities of the emerging Central Range—this concept is captured within our hypothesis H3 and H3A, which we find supported, in line with other recent publications (Table 4). Our hypothesis H3B assumed a temporal colonization sequence from west to the east based on some of the available geological data [75]. However, for the diving beetles, we roughly find the opposite pattern, with colonization occurring from east to west, and more in line with the broader patterns seen in the geological data available across New Guinea and consistent with tectonic models that show sinistral strike slip faulting along the northern margin of New Guinea occurring from east to west [5, 6, 18] (Fig. 6). Data for other taxa in general are scarce and highlights the need for strongly enhanced biological and geological sampling along the Central Range.

Our hypothesis H3C focused on the Papuan Peninsula. This part of New Guinea (sometimes referred to as East Papuan Composite Terrane EPCT, or Woodlark Plate) has been considered one of the oldest terrestrial areas of New Guinea, possibly existing as a separate island for since > 25 Ma (e.g., [50]). Indeed, several studies (Table 4) point to the Papuan Peninsula as an area of early diversification in New Guinea. We identify colonization events from the Central Range East towards the Papuan Peninsula from the mid Miocene, and then repeated interactions with the Central Range and north coast areas. (Fig. 6). The Bird’s Head might have been colonized earlier than expected based solely on geological evidence, but major lineage diversification appears to be more recent, from *ca*. 5 Ma (this study; [42]; Table 4). Here, studies more focused on the Bird’s Head, with comprehensive taxon sampling, remain a future task.

## Conclusion

Large scale diversification of New Guinea *Exocelina* is to a large extent structured geographically, the explanation of which lies in geological time and hints towards early lineage diversification in a proto Papuan archipelagic setting. Towards the tip nodes, processes took place across a more or less existing landscape. We highlighted the need for further studies, with comprehensive taxonomic and geographic sampling. Preliminary data for hyperdiverse weevils suggest that even smaller mountain ranges such as Cyclops can harbour large numbers of endemic species within a single regnus [76], but their biogeographic origins remain to be studied. Also, finer scale investigation using population genomic data as well as research on species’ dispersal ecology will help to untangle the processes that led to a high degree of local endemism of such beetles, in our case with closely related species often occurring close to each other such as in clade 7 with a species group endemic to mostly higher elevation of one mountain block (Figs. 4, 5; Additional file 1: Appendix 12).

## Methods

### Review of geological and biogeographic background

#### The north coast region

To a large extent, the northern part of New Guinea, including parts of the Central Range, are of Pacific affinity. This includes also the Bewani, Adelbert and Finisterre Mountains, parts of the Papuan Peninsula (see also below) as well as New Britain and parts of the Arfak Mountains, Yapen, Biak and Waigeo islands, among others (Fig. 1). Biologists have discussed the biogeographic significance of these elements of Pacific affinity, which has been referred to as the “Solomons Arc” (e.g., [28, 51]); summary in [33]. These authors interpreted this arc to represent an accreted island arc system that shows pronounced species level endemism, possibly derived from times when these terranes were islands adrift in the Pacific. This hypothesis implies an old origin of endemic lineages, as well as an initial stepping-stone dispersal from an Asian or a Pacific source area and then along an island arc with subsequent local speciation. This in turn requires that part of the island arc system was broadly exposed above sea-level for a considerable part of its history, e.g., since the mid-Miocene (15–11 Ma, [33] or earlier (> 30 Ma, [43]. We use this assumption as *hypothesis H1* as suggested in the introduction.

A very poorly known area of Pacific affinity is the Gauttier Terrane, with the Foja Mountains as its prominent feature. It largely consists of basaltic lavas and breccia (and potentially ultramafic rocks) overlain by carbonates and volcanic ash [3, 77]. Few geological data exist from this region, but it is considered to be related to the Torricelli Terrane to the east, that is a terrane of Pacific affinity thought to be part of an island arc that accreted to New Guinea’s north coast after the early Miocene (< 23 Ma). Colonization of the Foja Mountains could thus be a comparably old biogeographic event, and this relates to our *hypothesis H1A*.

#### The ultramafics

Collision of other material of Pacific affinity with the northern edge of New Guinea likely occurred earlier and is represented by the various ultramafic/ophiolite rocks found mainly across central and western New Guinea [6, 51] (Fig. 1). These ophiolite and ultramafic rocks consist of oceanic lithosphere pushed southward and upward on top of the existing New Guinea landmass (i.e., due to obduction). They are considered to have been emplaced onto the northern margin of New Guinea at different times and locations between the late Cretaceous and the early Miocene (i.e., 100 Ma to *ca.* 20 Ma) [3, 14]. These seafloor rocks were subsequently ‘sandwiched’ between the fold and thrust belt of New Guinea’s Central Range, as well as the northern accreted terranes of Pacific affinity (“Solomons Arc” discussed above) during the late Miocene (11–5 Ma) to present day. The ophiolite sequences include the Irian Ophiolites (in Papua Province, Indonesia), the April Ultramafics (western Papua New Guinea, PNG), and the (east) Papuan Ophiolite (Papuan Peninsula, PNG) [2, 3] and these broadly correspond to the “Papuan Arc” terrane of [51]. We note that there are other areas of similar geology in the Cyclops Mountains [78] and the Mount Gamey area north of the Weyland Mountains [79], however, limited geological data in these regions means that little is known about their provenance and tectonic history.

Pioneering biogeographic work by [51, 58] suggested that the “Papuan Arc” was adrift in the Pacific and gave rise to genus level endemism. The ophiolites were suggested to have also played a role as stepping-stones for Asian fauna on their way to present day New Guinea. This hypothesis is not strongly supported by geological evidence, as: (1) ophiolites by definition are sections of the seafloor and the upper mantle that have been thrust on to continental crust—this means that ophiolites found on landmasses today, were covered by > 1000 m of seawater prior to their obduction, and could not harbor terrestrial life, and (2) the New Guinea ophiolites certainly stem from Pacific areas remote from an Asian source area. Note that the younger volcanic arc rocks of Pacific affinity would have likely been submerged volcanoes or small volcanic islands prior to their collision with New Guinea (see [41]). However, for our present phylogenetic analysis of one genus-level radiation, we suggested *hypothesis H2*, i.e. that some of the older clades should occur in, or nearby, areas of ophiolite and ultramafic rocks (e.g., Mesozoic ultramafic rocks, Fig. 2).

#### Central Range, Bird’s Head and Papuan Peninsula

The spine of New Guinea is the 1300 km long and up to 150 km wide central highland chain, otherwise known as the Central Range, consisting of the major geographic features, Maoke, Bismarck and Owen Stanley Range. It stretches from the easternmost end of the Bird’s Head area (*ca.* 135°E) to *ca.* 145°E, which is the westernmost end of the Owen Stanley range on the Papuan Peninsula. The latter is the Bird’s tail and eastward extension of the Central Range. More than 20 summits lie above 4000 m altitude, and summits above 3000 m are common. It includes a major fold-and-thrust belt in the Central Range which represents the deformed passive margin of the Australian continent. Prominent features include the Snow and Star Mountains as well as the Papuan Peninsula. Available geological data indicates that uplift propagated westward along the northern section of Papua New Guinea (i.e., within the ‘Solomons Arc’ region) at 8–5 Ma—this was associated with the strike-slip motion at the plate boundary at this time [18]. The Central Range was uplifted within the last 5 Ma, with uplift propagating from the north towards the south [4, 18]. There is some evidence that indicates this uplift may have propagated westward from Papua New Guinea, into Papua at ~ 2–3 Ma [5, 13], as well as eastward (e.g., [75]), including rapid rates of uplift after 3 Ma in the Papuan Peninsula (e.g., the Dayman Dome: [80] [81]).

There is no doubt that New Guinea also records evidence of earlier tectonic events that would have driven uplift in different parts of the island (e.g., a model proposed by [82] suggested that an underthrusting of the Australian continent beneath an Inner Melanesian arc resulted in an orogeny restricted to eastern New Guinea (forming the Papuan Peninsula Orogen, Fig. 1, *ca.* 30–35 Ma). This conceptual model is supported by low temperature thermochronology data that shows rocks of the Müller Range underwent a phase of early Eocene to Oligocene cooling and interpreted to provide potential evidence of the initiation of plate collision and associated uplift [83]. This event, together with a drop in global sea level during the late Oligocene-early Miocene (e.g., [84, 85]) resulted in large areas of New Guinea being exposed above sea-level (e.g., [7, 75, 86]). However, much of New Guinea was submerged again during the mid-late Miocene (*ca.* 5–15 Ma) due to subsidence outpacing a continued decline of global sea level. The time and extent to which land began to emerge during and after this time ultimately depends on the interpretation of the available geological data.

One piece of evidence that has been used to explain the emergence of land during the Miocene are the rocks classified as the Makats Formation, found in the Mamberamo region north of the Central Range. These sedimentary rocks were most likely deposited in relatively deep water, but contain detritus, including metamorphic rocks that must have been sourced from exposed land [86]. There is no firm evidence to indicate whether the source of the material was from the north (e.g., island arcs) or the south (e.g., uplifted ‘Australian’ crust) [86]. Despite this, [75] proposed that the metamorphic detritus within the Makats Formation were most likely sourced from a > 500 km distant landmass that first emerged in the westernmost part of the Central Range. The timing of this emergence was originally reported by [75] *ca.* 16–14 Ma corresponding to the maximum depositional age of the Makats Formation. We revised this reported age using the planktonic foraminifera that occur within the Makats Formation (i.e., first reported by [86] together with the currently accepted age ranges for the diagnostic planktonic foraminifera within the Makats Formation using the Mikrotax database (http://www.mikrotax.org/pforams—last accessed 7 April 2020) [87]. Based on this, the Makats Formation was most likely deposited between *ca.* 13.4 Ma and *ca.* 10.5 Ma (and most certainly no younger than *ca.* 6.8 Ma, based on the oldest ages reported for the overlying Mamberamo Formation). Smaller islands may also have been present from *ca.* 15 Ma. This is consistent with low-temperature thermochronology data and thermal history modelling (e.g., [4, 18, 83]. The Central Range region was considered to have grown laterally and in height, gaining maximum elevations of up to 2000 m by 8 Ma, and 4000 m by 6 Ma [75]. Towards the east (Papua New Guinea, PNG), the Central Range orogeny appears to be younger [82]. The Central Range continued to grow as the fold-and-thrust belt rapidly developed from 5 Ma to present. This period of time also saw the formation of mountains associated with crustal stretching and metamorphic core complex development (e.g., the Wandamen Peninsula in West Papua [13] and the Dayman Dome of the Papuan Peninsula, e.g., [80, 81]). New Guinea’s emergence from the sea was progressive as there were multiple phases of uplift and submergence in western New Guinea over the past 5 Ma [7]. So, while sections of the Central Range were being uplifted from 8 Ma, other regions such as the Papuan Peninsula, were not uplifted until the Early Pliocene, and the final stage of uplift across the island likely occurred between 1 Ma and present day [7].

Some studies suggest an older age for the emergence of the area spanning the Central Range of eastern PNG and the Papuan Peninsula including Herzog Mountains, e.g., referring to that region as “Woodlark Plate” [42], or, more focussed on the Papuan Peninsula including Herzog Mountains, as “East Papuan Composite Terrane, EPCT” [28, 33, 50]. The area interpreted as the EPCT is a concept that was proposed by [77]. It is said to consist of a series of crustal fragments that were torn off the northern margin of New Guinea in the Cretaceous or earlier. These rocks supposedly accreted with other crustal fragments from the Pacific between 52 and 23 Ma, all of which later collided with the northern margin of New Guinea during the Miocene [77]. The EPCT formation could also be linked to what [82] describe as the Oligocene Peninsular orogeny (*ca.* 35–30 Ma). The remnants of the EPCT today span the modern-day Papuan Peninsula including the Herzog Mountains. Based on this conceptual tectonic model, some biogeographers have assumed that the EPCT may have formed islands or a landmass north of New Guinea before 25 Ma, or at least representing one of the oldest terrestrial habitats in the proto Papuan archipelago (e.g., [23, 33, 42, 45, 48, 51]). In such conceptual models, the EPCT (and “Woodlark Plate” in [42] are proposed to harbor old biota and serve as source area for other parts of New Guinea.

However, the geological data on which the concept of the EPCT was formed simply indicates that a body of water developed between rocks of Australian Plate affinity along the northern margin of New Guinea [77]. It is unclear: (1) how wide this gap was; (2) whether all land connections were severed, and (3) whether this body of water was an ocean, or a rift valley that was subsequently inundated during higher sea levels. Available geological data does not indicate whether the EPCT was or was not exposed above sea-level before its final accretion to the northern margin of New Guinea during the Miocene. Considering these points, there is a high level of uncertainty as to whether the EPCT existed, and if it did, to what extent it may have been an emergent landmass within the Pacific Ocean prior to the middle to late Miocene. Rather than interpreting our data using a highly uncertain, conceptual tectonic model, we instead focus on whether there is any difference in which species are found within the different terrane affinities in the Papuan Peninsula, as well as the evidence that indicates the Papuan Peninsula was uplifted from the Miocene or later. To allow comparisons between biogeographic studies, in this paper, the rocks that others recognize as belonging to the EPCT correspond with the region marked by the East Papuan Ophiolite (Fig. 1). Similarly, the concept of the “Woodlark Plate” and its long-term biogeographic significance as discussed in [42] is highly problematic. This work used a map of the modern-day plate boundaries [88], where plate boundaries have largely been drawn on the basis of earthquake locations and GPS velocity data that suggest parts of the Earth’s crust are moving as a cohesive plate. Therefore, this does not outline entities for historical analyses that span millions of years.

Our *hypothesis H3* relates to that, suggesting an early diversification on the present Central Range, possibly in an initial setting of a chain of islands, and subsequent colonization of surrounding areas such as the Bird’s Head and the Papuan Peninsula. This scenario was in part tested by [41], who suggested recent colonization of the Bird’s Head and Papuan Peninsula’s out of the Central Range, and [45] who found a colonization sequence from craton and later Papuan Peninsula (EPTC), to the Bird’s Head region and then areas with Pacific affinity.

We test this further under the following assumptions: (*hypothesis H3A*) the 1300 km long Central Range initially consisted of several islands, this implies localized radiations in different present-day highland blocks; (*hypothesis H3B*) there is a temporal sequence from west to the east; (*hypothesis H3C*) mountains of eastern PNG have existed as a separate island before 25 Ma and do therefore harbor fauna older than expected based on the above geological scenarios, and served as source area for other parts of New Guinea.

### Assembly of the geological map

The geological terrane map that is shown in Fig. 2, and online at https://arcg.is/189zmz, is a simplification of numerous 1:250,000 scale geological maps of Indonesian New Guinea (currently Papua and West Papua Provinces, previously known as “Irian Jaya”) and Papua New Guinea. The Indonesian maps were developed by the Indonesian Geological Research and Development Centre (GRDC) (Pusat Penelitian dan Pengembangan Geologi (Indonesia)) and the Australian Bureau of Mineral Resources (BMR), now known as Geoscience Australia. The Papua New Guinea maps were developed by the Papua New Guinea Mineral Authority as well as the Australian Geological Survey Organisation (AGSO), now known as Geoscience Australia (for map sources and methods, see Additional file 1: Appendix 1).

Digital scans of the Indonesian New Guinea geological maps were orthorectified in ArcGIS v10.4 using the WGS84 datum. Each geological unit was mapped as a separate polygon and was assigned metadata according to the map rubric. Each polygon was classified according to one of seven geological terranes (Table 1). For Papua New Guinea, GIS maps were purchased from the *PNG Mineral Authority* (PNG_Geol250, 2002) and these were classified according to the same seven geological terranes as were used for Indonesian New Guinea. Some minor editing of the polygons was made to cut the digitized polygons to fit the current coastline. While every effort was made to quality check the data, readers should note that the digitization and reclassification involved individually modifying the attributes of > 20,000 polygons. Those polygons with a common terrane attribute and shared boundary were later merged to reduce the size of the datafile. Considering the size and complexity of the geological map, we expect that there will be minor errors. Readers should also note that the geological boundaries that we present have been digitized from hardcopy paper maps. There is some component of uncertainty associated with the original hardcopy maps. For instance, the regions of adjoining map sheets show considerable differences in the extent or continuity of particular rock types, particularly at the international border between Indonesia and PNG. Also, most of the maps were drawn before GPS was widely available, meaning there is an issue with the true location of the base maps that were used as well as the geologist’s ability to locate themselves on the map. Those who produced the PNG_Geol250 (2002) data estimated the geological boundaries in the digital dataset have an accuracy between 250 m (1 mm at 1:250,000) and 3.75 km (about 1.5 cm at 1:250,000 map scale), with the uncertainty being greatest in the highland regions and at the edge of adjoining map sheets. The Irian Jaya series maps likely have a similar level of spatial uncertainty. This does not account for uncertainty associated with distortion of the original paper maps before or during scanning. Having said this, readers should also note that the map presented in Fig. 2 is a much more detailed general terrane map of New Guinea compared to what is presented in earlier geological review papers (e.g., [2, 3]), and more importantly, the map is a much more accurate representation of the geology of New Guinea compared to the maps used in most existing biogeography papers.

### Geospatial analysis

The latitude, longitude and altitudinal data obtained from a handheld GPS unit at each sample site were compiled in a database leading to 638 observations at 303 mapped localities. For instances where a reliable altitude could not be obtained in the field (e.g., due to dense tree cover), we assigned a value based on the sample site position and the corresponding cell from a digital elevation model (constructed from the ETOPO1 Bedrock Global Relief Model: [89]. Each database entry was classified with a number between one to five to represent the altitudinal range of each sampling site (< 500 m; 500–1000 m, 1000–1500 m, 1500–2000 m and > 2000 m), as well as a number between one and five to represent the geological terrane that each sampling site was encapsulated by (using the values recorded in Additional file 2: Appendix 2). The numerical value for the altitude and terrane was required to perform the Grouping Analysis tool (discussed below). Each database entry was double-checked as there were several instances where a sample site lay near a boundary of two terranes. In such instances, a decision was made to retain this classification, or to assign it to another terrane. This decision was most commonly employed when sample sites were located near the boundary between ‘Australian Plate affinity’ and ‘Pacific Plate affinity’ and were instead reassigned to the ‘Transition’ classification.

A one-to-many database join was used to assign the latitude, longitude, altitude and geological terrane to each identified species. Each database entry was also assigned a unique value within the database (Additional file 1: Appendix 2).

The Grouping Analysis tool, part of the ArcGIS Spatial Analysis toolbox extension was used to explore potential spatial relationships within the database. This technique utilizes unsupervised machine learning methods to determine natural groupings within a dataset. This technique is considered unsupervised because it does not require a set of pre-classified features to guide or train the algorithm that determines groupings within a spatial database. While our ultimate aim of employing this technique was to determine if there are spatial patterns within our database, we did not apply a spatial constraint to the algorithm, which means that the algorithm assumes there is no spatial correlation between any two sample locations. This means that if a spatial pattern is identified within the analysis, it is dependent on the altitude or underlying geology (or both). The tool requires the user to specify how many populations between 2 and 15 might exist within the dataset. After running the algorithm using different input parameters, we limited the final output to five populations. Readers should also note that it is possible that the numerical value assigned to represent the altitudinal range and geological terrane might also influence the results determined by this algorithm. Considering these limitations, we used the tool for data exploration purposes only, using it to test if any apparent relationship could be identified between species and altitude, species and underlying geology and species plus both altitude and geology.

### Taxon sampling and molecular biology

We build upon the recent molecular framework of [41, 46], and added data from 41 additional species of *Exocelina* diving beetles compared to the most recent molecular treatment of the genus [46]. Most of the data sequenced for these new taxa was derived from older museum specimens. Complete genomic DNA was extracted with a Qiagen DNeasy Blood & Tissue kit (Hilden, Germany) using head and pronotum, or entire beetles. Using PCR protocols described in [41], we amplified and sequenced fragments of the following genes; mitochondrial cytochrome c oxidase I (cox1), cytochrome c oxidase II (cox2) and cytochrome b (cob), in addition to the nuclear histone 3 (H3), histone 4 (H4), 18S rRNA (18S), Carbomoylphosphate synthase (CAD) and Alpha-Spectrin (Asp). All new sequences will deposited in GenBank after publication of their formal names in [90, 91] so that new data can be uploaded with their final names. The matrix for this project has been deposited in Dryad: https://doi.org/10.5061/dryad.ns1rn8prj.

### Alignment and phylogenetic inference

The resulting sequences were edited in Geneious R11 (Biomatters, USA) and checked for sequencing errors. Once assembled, the consensus sequences were aligned using MUSCLE [92] with existing datasets [41, 46] as well as a selection of outgroups chosen from the comprehensive phylogeny of [93] to facilitate the use of secondary calibrations in the BEAST divergence time analyses (see below). The resulting gene fragment alignments were checked for stop codons or indels and concatenated to produce a final matrix comprising 237 specimens (including 205 *Exocelina* specimens), and 4226 aligned nucleotides.

The concatenated matrix was analyzed in a maximum likelihood framework using IQ-TREE 1.6.6 [94]. The matrix was a priori subdivided into non-coding gene fragments and codon positions of coding gene fragments for a total of 22 partitions. The optimal partitioning scheme and corresponding models of nucleotide substitutions were searched simultaneously using ModelFinder [95] as implemented in IQTREE 1.6.6 among all available models and selected using the corrected Akaike Information Criterion (AICc) (see Additional file 1: Appendix 3 for the resulting partitioning scheme and models used in the IQ-TREE analyses). We performed 500 tree searches to avoid local optima. For each tree search, we performed branch support calculations with 1000 ultrafast bootstrap replicates (UFBoot, [96, 97] and 1000 SH-aLRT tests [98]. We used the hill-climbing nearest-neighbor interchange topology search strategy implemented in IQ-TREE to avoid severe model violations leading to biased ultrafast bootstrap estimations [97].

### Divergence time estimation

There is no fossil known of the genus *Exocelina*, and the fossil record within Copelatinae is very scarce. Previous attempts at estimating absolute divergence times across Copelatinae have suggested different hypotheses pertaining to the temporal evolution of these beetles [41, 46, 65]. These discrepancies are largely linked to alternative calibration strategies. Recently, a new fossil-based dated phylogenetic framework for diving beetles has been developed based on the phylogeny of [66]. We rely on multiple secondary calibrations from this study to calibrate clocks and estimate divergence times. Specifically, we constrained the split between Dytiscinae and closely related subfamilies Copelatinae, Cybistrinae and Laccophilinae (i.e., the stem of Dytiscinae in [66] and the root of the tree in the current study) with a uniform distribution encompassing the 95% credibility interval recovered for this node in [66] (i.e., 122–141.4 Ma). We enforced the sister relationships between Laccophilinae and Cybistrinae in the BEAST analyses following [66]. We also constrained the crown of Cybistrinae (95% HPD = 37.4–81.5), crown of Laccophilinae (95% HPD = 44.7–98.3), crown of Copelatinae (95% HPD = 58–114.2), crown Cybistrinae + Laccophilinae (95% HPD = 71.6–120.6) and crown of Copelatinae + Cybistrinae + Laccophilinae (95% HPD = 88.8–135.5) with uniform prior distributions matching the 95% credibility intervals recovered in [66].

We used PartitionFinder 2 with the *greedy* algorithm, linked branch lengths and the set of models included in the program BEAST 1.10.4 [99], to select the optimal partitioning scheme and models of nucleotide substitution using the same 22 initial partitions as in the IQ-TREE analyses. The partitions and corresponding models of nucleotide substitution were selected with the Bayesian Information Criterion. We either assigned one clock to the mitochondrial partitions and another to the nuclear partitions (2 clocks in total), or a different clock to each partition recovered in PartitionFinder (12 clocks in total, see Additional file 1: Appendix 4 for the best partitioning scheme and models used in the BEAST analyses). We used relaxed molecular clocks with uncorrelated rates drawn from a lognormal distribution in BEAUti 1.10.4 [99]. The *Tree Model* was selected as Yule or birth death in different analyses. All other parameters were left to default. The analyses were conducted in BEAST 1.10.4 with 100 million generations, a parameter and tree sampling every 5,000 generations, and estimation of marginal likelihood using path-sampling and stepping-stone sampling with default parameters (*chainLength* = 1,000,000; *pathSteps* = 100; *α* = 0.3). The best scoring IQ-TREE topology out of 500 independent ML tree searches was constrained as a fixed input tree (with the unique modification being the enforced sister relationship between Laccophilinae and Cybistrinae) by manually editing the BEAUti.xml file. The four different analyses were compared based on their marginal likelihood estimates (MLE), and the one with the lowest MLE was used for further analyses.

### Ancestral state reconstructions

We used the Bayesian Binary MCMC (BBM) method as implemented in RASP 4.2 [100] to estimate ancestral ranges across the New Guinean *Exocelina* radiation. To perform the reconstructions, we used the best BEAST maximum credibility clade tree based on MLE comparisons (Table 2). We coded each taxon with geology and geography (Additional file 1: Appendix 2). For altitude and geology, beetles were assigned to different categories using the geospatial model we compiled for this project. The altitude was coded using five discrete categories: (1) 0–500 m, (2) 501–1000 m, (3) 1001–1500 m, (4) 1501–2000 m, (5) above 2000 m. These intervals are subjective but provide the framework to visualize the three-dimensional distribution of the beetles. While we provide an outline of the elevational evolution of New Guinea in the introduction, note that it is currently not possible to suggest a reliable paleoaltimetric model for the island. Uplift was comparably fast with a rate of up to 10 mm/year^**−1**^ for some areas (e.g., the Wandamen Peninsula: [13] and up to 51 mm/year^**−1**^ for others (e.g., 17–51 mm/year^**−1**^ D’Entrecasteaux Island; [101]), at least from 3 Ma to the present. This might also apply for (parts of the) Central Range, which has seen rapid uplift in the past 5 Ma. But the rate of uplift might vary over time and region, and the supposed submergence of vast areas would add additional uncertainty. The geology was coded based on our geological terrane map of New Guinea. For the coding, the three principal terranes were in part subdivided as follows: Northern belt into: “accreted Pacific Plate affinity” and “ultramafic”; the transition belt into:”Post Collisional Volcanics” and “Transition”, and gondwanan material as “Uplifted Australian Plate affinity” (see introduction).

The geographic coding does not consider the geological history but looks at the existing island and its major regions (https://arcg.is/189zmz “New Guinea Regional Classification”). We here differentiate between six areas: the north coast mountain ranges including Wandamen (coded as A), the Bird’s Head including its satellite islands (B), the Bird’s Neck (Lengguru) (G), the Papuan Peninsula including the Herzog Mountains to its north east (C), as well as three blocks of the Central Range (from the Weyland/Paniai region in the west up to Baliem Valley at *ca.* 139° E (coded as D), from Baliem valley east to the Star Mountains including in Sandaun Province of PNG 142° E (coded as E), and finally the mountain block east of Sandaun to 145° E) (coded as F). This geographic delineation is simplified and meant to possibly reveal large scale biogeographic patterns and is not based on previously suggested New Guinea areas of endemism (e.g., [102]. It is also important to note that the delineation of the geographic areas is subjective to some degree; for example, in the west of area C, mainly Papuan Peninsula, we include the Herzog Mountains (that could alternatively be assigned to “A”, north coast ranges). The single species from the Bird’s Neck (Lengguru: Kaimana) region was coded in its own region (G). Alternatives would be assigning the Bird’s Neck to the Bird’s Head region (B) or the Central Range West (D) (Additional file 1: Appendix 11). Parts of the central range northern slopes, that are geologically also of Pacific affinity (Figs. 2, 4) were coded as geographically belonging to the Central Range.

All analyses were conducted in a Bayesian framework in RASP 4.2. using a Markov chain Monte Carlo method with 10 chains running for 1 million generation and with a sampling every 1,000 generations. We used the estimated F81 model for all runs. The rest of options were left to default in BBM.

### Diversification rate dynamics estimation

We estimated diversification rate dynamics within *Exocelina* using the program BAMM 2.5.0 [103]. The analyses were performed with four reversible jump MCMC running for 1 million generations and sampled every 1000 generations. Parameter priors were estimated in R using the *setBAMMpriors* function (*expectedNumberOfShifts* = 1.0; *lambdaInitPrior* = 0.800; *lambdaShiftPrior* = 0.067; *muInitPrior* = 0.800). We used different priors (0.1, 1, 2 and 5) for the parameter controlling the compound Poisson process that determines the prior probability of a rate shift along branches of the chronogram. Our taxon sampling comprises 142 species of the New Guinean radiation (described and undescribed ones), yet we hypothesize that the extant species richness of the New Guinean radiation is closer to *ca.* 190 species. Therefore, the global sampling fraction was setup to 0.75 (New Guinean radiation only) in the different analyses. This is arguably more realistic than relying on the current described diversity (relying on the latter did not affect the results, data not shown). The BAMMoutput files were analyzed using BAMMtools 2.1.6 [104]. The posterior distribution of the BAMM analysis was used to estimate the best shift configuration and the 95% credible set of distinct diversification models.

We also tested the fit of various diversification dynamics scenarios to the entire New Guinean *Exocelina* diving beetle radiation. We relied upon constant‐rate, time‐dependent and diversity-dependent models of diversification as implemented in a maximum‐likelihood framework. The different models were fitted using the fit_bd function rom the R package RPANDA 1.8 [105] and the *dd_ML* function from the R-package DDD 4.3 [106], (see i.e., [107] for more details). Missing taxon sampling at the species level was also taken into account, using a global fraction of the expected species richness in the genus (i.e., 142/190 = *ca.* 0.75).

We tested the fit of the following models: (1) speciation rate constant through time with no extinction (BCST), (2) speciation and extinction rates constant through time (BCSTDCST), (3) speciation rate varying exponentially through time with no extinction (BtimeVarEXPO), (4) speciation rate varying linearly through time with no extinction (BtimeVarLIN), (5) speciation rate varying exponentially through time with constant extinction (BtimeVarDCSTEXPO), (6) speciation rate varying linearly through time with constant extinction (BtimeVarDCSTLIN), (7) extinction rate varying exponentially through time with constant speciation (BCSTDtimeVarEXPO), (8) extinction rate varying linearly through time with constant speciation (BCSTDtimeVarLIN), (9) speciation and extinction rates varying exponentially through time (BtimeVarDtimeVarEXPO), (10) speciation and extinction rates varying linearly through time (BtimeVarDtimeVarLIN), (11) speciation rate varying linearly with diversity without extinction (DDL), (12) speciation rate varying linearly with diversity with constant extinction (DDL + E), (13) speciation rate varying exponentially with diversity with constant extinction (DDX + E), (14) speciation and extinction rates varying linearly with diversity (DDL + EL).

In the time-dependent and diversity-dependent models, speciation and extinction rates (respectively λ and μ) could vary as a continuous function of time or diversity. This function was assumed to be either linear or exponential. The parameters α and β measure the sign and rapidity of time-variation for respectively speciation and extinction rates. Positive values of α or β can be interpreted as an indicator of speciation or extinction slowdown, while negative values indicate an acceleration of speciation or extinction. The parameter K measures the carrying-capacity in diversity-dependent models. These 14 models were compared with the AICc and ΔAIC to determine best-fit to the time-calibrated phylogeny.

## Supplementary Information

**Additional file 1: Appendix 1.** Methodology for the construction of the New Guinea geological terrane map including references for map building. **Appendix 2.** Separate Excel file containing the geospatial data. **Appendix 3.** Partitioning scheme and models of nucleotide substitution for IQ-TREE ML tree searches as selected under ModelFinder. **Appendix 4.** Partitioning scheme and models of nucleotide substitution for BEAST divergence time estimation as selected using PartitionFinder. **Appendix 5.** Best scoring IQ-TREE ML tree (SH-aLRT/UFboot at nodes). **Appendix 6.** Best scoring IQ-TREE ML tree in newick format. **Appendix 7.** Best BEAST analysis chronogram with median ages. **Appendix 8.** Best BEAST analysis chronogram with median ages in newick format. **Appendix 9.** Spatial analysis map. **Appendix 10.** Heat maps for geology, altitude and geospatial analysis groups and projected onto the phylogenetic hypothesis. **Appendix 11.** Detailed outputs of BMM analyses. **Appendix 12.** Altitudinal distribution map. **Appendix 13.** Results of BAMM with a prior of 0.1.

**Additional file 2.** Separate Excel file containing Appendix 2, summary of the geospatial data.

## Data Availability

References to the data generated or analyzed during this study are included in this published article. The sequence data are deposited in Dryad: https://doi.org/10.5061/dryad.ns1rn8prj. The geospatial data are online under: https://arcg.is/189zmz. CETAF user statement: „Data on genetic material contained in this taxonomic publication are published for non-commercial use only. Utilization by third parties for purposes other than non-commercial scientific research may infringe the conditions under which the genetic resources were originally accessed, and should not be undertaken without obtaining consent from the original provider of the genetic material.”

## Abbreviations

HPD: Highest posterior density
Ma: Million years ago
rRNA: Ribosomal RNA
