# Supplementary material for: New Guinean orogenic dynamics and biota evolution revealed using a custom geospatial analysis pipeline
Source: BMC Ecol Evol. 2021 Apr 6;21:51. doi: 10.1186/s12862-021-01764-2 (PMC8022562; doi:10.1186/s12862-021-01764-2)
Supplement: Supplementary file 1 — Additional file 1: Appendix 1. Methodology for the construction of the New Guinea geological terrane map including references for map building. Appendix 2. Separate Excel file containing the geospatial data. Appendix 3. Partitioning scheme and models of nucleotide substitution for IQ-TREE ML tree searches as selected under ModelFinder. Appendix 4. Partitioning scheme and models of nucleotide substitution for BEAST divergence time estimation as selected using PartitionFinder. Appendix 5. Best scoring IQ-TREE ML tree (SH-aLRT/UFboot at nodes). Appendix 6. Best scoring IQ-TREE ML tree in newick format. Appendix 7. Best BEAST analysis chronogram with median ages. Appendix 8. Best BEAST analysis chronogram with median ages in newick format. Appendix 9. Spatial analysis map. Appendix 10. Heat maps for geology, altitude and geospatial analysis groups and projected onto the phylogenetic hypothesis. Appendix 11. Detailed outputs of BMM analyses. Appendix 12. Altitudinal distribution map. Appendix 13. Results of BAMM with a prior of 0.1. [file 12862_2021_1764_MOESM1_ESM.docx]

**Additional File 1**

**New Guinean orogenic dynamics and biota evolution revealed using a custom geospatial analyses pipeline**

**Appendix 1**

Methodology for the construction of the New Guinea geological terrane map including references for map building.

**Appendix 2**

Separate Excel file containing the geospatial data in additional file 2.

**Appendix 3**

Partitioning scheme and models of nucleotide substitution for IQ-TREE ML tree searches as selected under ModelFinder

**Appendix 4**

Partitioning scheme and models of nucleotide substitution for BEAST divergence time estimation as selected using PartitionFinder

**Appendix 5**

Best scoring IQ-TREE ML tree (SH-aLRT / UFboot at nodes)

**Appendix 6**

Best scoring IQ-TREE ML tree in newick format

**Appendix 7**

Best BEAST analysis chronogram with median ages

**Appendix 8**

Best BEAST analysis chronogram with median ages in newick format

**Appendix 9**

Spatial analysis map

**Appendix 10**

Heat maps for geology, altitude and geospatial analysis groups and projected onto the phylogenetic hypothesis.

**Appendix 11**

Detailed outputs of BMM analyses

**Appendix 12**

Altitudinal distribution map

**Appendix 13**

Results of BAMM with a prior of 0.1.

**Appendix 1**

Methodology for the construction of the New Guinea geological terrane map including references for map building.

| The geological terrane map that is shown in Figure 2 is a simplification of numerous 1:250,000 scale geological maps of Indonesian New Guinea (previously known as “Irian Jaya”) and Papua New Guinea. The Indonesian maps were developed by the Indonesian Geological Research and Development Centre (GRDC) (Pusat Penelitian dan Pengembangan Geologi (Indonesia)) |
| --- |

and the *Australian Bureau of Mineral Resources* (BMR), now known as *Geoscience Australia*. The Papua New Guinea maps were developed by the *Papua New Guinea Mineral Authority* as well as the *Australian Geological Survey Organisation* (AGSO), now known as *Geoscience Australia*. The references for the maps that were used to produce this compilation are provided in the references list below.

*Methodology*

Digital scans of the Indonesian New Guinea geological maps were orthorectified in ArcGIS using the WGS84 datum. Each geological unit was mapped as a separate polygon and was assigned metadata according to the map rubric. Each polygon was classified according to one of nine geological terranes (Suppl. Table 1). For Papua New Guinea, GIS maps were purchased from the *PNG Mineral Authority* (PNG_Geol250, 2002) and these were classified according to the same nine geological terranes as were used for Indonesian New Guinea. Some minor editing of the polygons was made to minimize issues associated with the edge of the original map sheets or to cut the digitized polygons to fit the current coastline.

While every effort was made to quality check the data, readers should note that the digitization and reclassification involved individually modifying the attributes of >20,000 polygons. We therefore expect that there will be minor errors in the dataset. Readers should also note that the geological boundaries that we present have been digitised from hardcopy paper maps. There is some component of uncertainty associated with the original hardcopy maps. For instance, all of the maps were drawn before GPS was widely available, meaning there is an issue with the true location of the base maps that were used as well as the geologist’s ability locate themselves on the map. Those who produced the PNG_Geol250 (2002) data estimated the geological boundaries in the digital dataset have an accuracy between +/- 250m (1 mm at 1:250,000) and 3.75 km (about 1.5 cm at 1:250000 map scale), with the uncertainty being greatest in the highland regions. The Irian Jaya series maps likely have a similar level of spatial uncertainty. This does not account for uncertainty associated with distortion of the original paper maps before or during scanning. Having said this, readers should also note that what is presented in Figure 2 is a much more detailed general terrane map of New Guinea compared to what is presented in earlier geological review papers (e.g., Davies 2012; Baldwin et al., 2012), and more importantly, the map is a much more accurate representation of the geology of New Guinea compared to most existing biogeography papers.

**References for map building**

The map sheets that were used in the process of compiling the regional geological terrane map are listed below:

Amri, P., Sanyoto, B., Hamonangan, B., Supriatna, S., Simanjuntak, W. and Pieters, P.E. 1990. Geological map of the Sorong sheet, Irian Jaya, Scale 1:250000. Geological Research and Development Centre, Bandung, Indonesia.

Atmawinata, S., Hakim, A.S., Pieters, P.E. 1989. Geological map of the Ransiki sheet, Irian Jaya, Scale 1:250000. Geological Research and Development Centre, Bandung, Indonesia.

Atmawinata, S., Ratman, N., Pieters, P.E. 1989. Geological map of the Yapen sheet, Irian Jaya, Scale 1:250000. Geological Research and Development Centre, Bandung, Indonesia.

Gafoer, S., Budhitrisna, T., 1995. Geological map of the Sarmi and Bufareh sheets, Irian Jaya, Scale 1:250000. Geological Research and Development Centre, Bandung, Indonesia.

Hakim, A.S., Harahap, B.H. 1994. Geological map of the Waren Quadrangle, Irian Jaya, Scale 1:250000. Geological Research and Development Centre, Bandung, Indonesia.

Hakim, A.S., Baharuddin, Susanto, E. 1995. Geological map of the Gunung Doom quadrangle, Irian Jaya, Scale 1:250000. Geological Research and Development Centre, Bandung, Indonesia.

Harahap, B.H., Hakim, A.S., Dow, D.B., 1990. Geological map of the Enaratoli sheet, Irian Jaya, Scale 1:250000. Geological Research and Development Centre, Bandung, Indonesia.

Harahap, B.H., Noya, Y. 1995. Geological map of the Rotanburg (Idenburg Barat) quadrangle, Irian Jaya, Scale 1:250000. Geological Research and Development Centre, Bandung, Indonesia.

Hartono, U., Amri, C.H., Pieters, P.E. 1989. Geological map of the Mar sheet, Irian Jaya, Scale 1:250000. Geological Research and Development Centre, Bandung, Indonesia.

Heryanto, R., Margono, U. 1995. Geological map of the Yapero & Birufu sheet, Irian Jaya, Scale 1:250000. Geological Research and Development Centre, Bandung, Indonesia.

Heryanto, R., Panggabean, H. 1995., Geological map of the Merauke sheet, Irian Jaya, Scale 1:250000. Geological Research and Development Centre, Bandung, Indonesia.

Heryanto, R., Panggabean, H., 1995. Geological map of the TG. Vals & Komolom sheet, Irian Jaya, Scale 1:250000. Geological Research and Development Centre, Bandung, Indonesia.

Koswara, A., 1995. Geological map of the Taritatu (Kerom) quadrangle, Irian Jaya, Scale 1:250000. Geological Research and Development Centre, Bandung, Indonesia.

Masria, M., Ratman, N., Suwitodirdjo, K., 1981. Geologic map of the Biak Quadrangle, Irian Jaya, Scale 1:250000. Geological Research and Development Centre, Bandung, Indonesia.

Panggabean, H., Pigram, C.J. 1989. Geological map of the Waghete sheet, Irian Jaya, Scale 1:250000. Geological Research and Development Centre, Bandung, Indonesia.

Panggabean, H. 1990. Geological map of the Omba sheet, Irian Jaya, Scale 1:250000. Geological Research and Development Centre, Bandung, Indonesia.

Panggabean, H., Amiruddin, Kusnama, Sutisna, K., Situmorang, R.L., Turkandi, T., Hermanto, B. 1995. Geological map of the Beoga quadrangle, Irian Jaya, Scale 1:250000. Geological Research and Development Centre, Bandung, Indonesia.

PNG_Geol250, 2002. Geological Survey of Papua New Guinea, Department of Mining, Independent State of Papua New Guinea. July 1, 2002

Ratman, N., Robinson, G.P., Pieters, P.E. 1989. Geological map of the Manokwari sheet, Irian Jaya, Scale 1:250000. Geological Research and Development Centre, Bandung, Indonesia.

Rusmana, A., Hartono, U., Pigram, C.J. 1989. Geological map of the Misool sheet, Irian Jaya, Scale 1:250000. Geological Research and Development Centre, Bandung, Indonesia.

Rusmana, E., Parris, K., Sukanta, U., Samodra, H., 1995. Geological map of the Timika quadrangle, Irian Jaya, Scale 1:250000. Geological Research and Development Centre, Bandung, Indonesia.

Suwarna, N. 1995. Geological map of the Mapi sheet, Irian Jaya, Scale 1:250000. Geological Research and Development Centre, Bandung, Indonesia.

Suwarna, N. Amin, T.C., 1995. Geological map of the Sarabih quadrangle, Irian Jaya, Scale 1:250000. Geological Research and Development Centre, Bandung, Indonesia.

Surono, S., Bachri, S., Bawono, S., Sukarana, D., 1995. Geological map of the Sawai sheet, Irian Jaya, Scale 1:250000. Geological Research and Development Centre, Bandung, Indonesia.

Sukanta, U., Wiryosujono, S., Hakim, A.S. 1995. Geological map of the Wamena quadrangle, Irian Jaya, Scale 1:250000. Geological Research and Development Centre, Bandung, Indonesia.

Suwarna, N., Kusnama, 1995. Geological map of the Muting quadrangle, Irian Jaya, Scale 1:250000. Geological Research and Development Centre, Bandung, Indonesia.

Sudana, D., Suwarna, N. 1995. Geological map of the Tanahmerah quadrangle, Irian Jaya, Scale 1:250000. Geological Research and Development Centre, Bandung, Indonesia.

Soetrisno, Amiruddin, 1995. Geological map of the Oksibil quadrangle, Irian Jaya, Scale 1:250000. Geological Research and Development Centre, Bandung, Indonesia.

Sidarto, Hartono, U., 1995. Geological map of the Jayawijaya quadrangle, Irian Jaya, Scale 1:250000. Geological Research and Development Centre, Bandung, Indonesia.

Suparman, M., Robinson, G.P., 1990. Geological map of the Fak Fak sheet, Irian Jaya, Scale 1:250000. Geological Research and Development Centre, Bandung, Indonesia.

Supriantna, S., Hakim, A.S., Apandi, T. 1995. Geological map of the Waigeo sheet, Irian Jaya, Scale 1:250000. Geological Research and Development Centre, Bandung, Indonesia.

Sukanta, U., Pigram, C.J. 1989. Geological map of the Taminabuan sheet, Irian Jaya, Scale 1:250000. Geological Research and Development Centre, Bandung, Indonesia.

Suwarna, N., Noya, Y. 1995. Geological map of the Jayapura (Peg. Cycloops) quadrangle, Irian Jaya, Scale 1:250000. Geological Research and Development Centre, Bandung, Indonesia.

Tobing, S.L., Robinson, G.P., Ryburn, R.J. 1990. Geological map of the Kaimana sheet, Irian Jaya. Scale 1:250000. Geological Research and Development Centre, Bandung, Indonesia.

Tobing, S.L., Achdan, A., Robinson, G.P., Ryburn, R.J. 1990. Geological map of the Steenkool sheet, Irian Jaya. Scale 1:250000. Geological Research and Development Centre, Bandung, Indonesia.

Tobing, S.L., Robinson, G.P. 1990. Geological map of the Palau Karas / Palau Adi sheet, Irian Jaya, Scale 1:250000. Geological Research and Development Centre, Bandung, Indonesia.

**Appendix 2**

Separate Excel file containing the geospatial data

**Appendix 3**

Partitioning scheme and models of nucleotide substitution for IQ-TREE ML tree searches as selected under ModelFinder

#nexus

begin sets;

charset 18S = 1-545;

charset ASPp1 = 546-1337\3;

charset ASPp2 = 547-1337\3;

charset ASPp3 = 548-1337\3;

charset CADp1 = 1338-2165\3;

charset CADp2 = 1339-2165\3;

charset CADp3 = 1340-2165\3;

charset CO1_pos1 = 2166-2897\3;

charset CO1_pos2 = 2167-2897\3;

charset CO1_pos3 = 2168-2897\3;

charset CO2_pos1 = 2898-3449\3;

charset CO2_pos2 = 2899-3449\3;

charset CO2_pos3 = 2900-3449\3;

charset COB_pos1 = 3450-3755\3;

charset COB_pos2 = 3451-3755\3;

charset COB_pos3 = 3452-3755\3;

charset H3_pos1 = 3756-4070\3;

charset H3_pos2 = 3757-4070\3;

charset H3_pos3 = 3758-4070\3;

charset H4_pos1 = 4071-4226\3;

charset H4_pos2 = 4072-4226\3;

charset H4_pos3 = 4073-4226\3;

charpartition mymodels =

TIMe+R3: 18S,

GTR+F+R4: ASPp1,

TPM3u+F+R3: ASPp2,

TN+F+I+G4: ASPp3,

TIM2+F+R2: CADp1,

TN+F+I: CADp2,

TIM2+F+I+G4: CADp3,

GTR+F+R4: CO1_pos1,

GTR+F+R2: CO1_pos2,

GTR+F+R5: CO1_pos3,

TIM2+F+R3: CO2_pos1,

HKY+F+R2: CO2_pos2,

GTR+F+I+G4: CO2_pos3,

TIM2+F+R4: COB_pos1,

TN+F+R3: COB_pos2,

GTR+F+R5: COB_pos3,

TN+F+G4: H3_pos1,

TIM2e+I: H3_pos2,

GTR+F+G4: H3_pos3,

GTR+F+G4: H4_pos1,

JC: H4_pos2,

TN+F+I: H4_pos3;

**Appendix 4**

Partitioning scheme and models of nucleotide substitution for BEAST divergence time estimation as selected using PartitionFinder

#nexus

begin sets;

charset Subset1 = 1-545;

charset Subset2 = 546-1337\3 1338-2165\3;

charset Subset3 = 547-1337\3 1339-2165\3 4073-4226\3 2167-2897\3;

charset Subset4 = 548-1337\3;

charset Subset5 = 1340-2165\3;

charset Subset6 = 2166-2897\3 3450-3755\3 2898-3449\3;

charset Subset7 = 2168-2897\3 2900-3449\3;

charset Subset8 = 3451-3755\3 2899-3449\3;

charset Subset9 = 3452-3755\3;

charset Subset10 = 3756-4070\3;

charset Subset11 = 4072-4226\3 3757-4070\3;

charset Subset12 = 3758-4070\3 4071-4226\3;

charpartition PartitionFinder = TRNEF+I+G:Subset1, GTR+I+G+X:Subset2, HKY+I+G+X:Subset3, HKY+I+G+X:Subset4, GTR+I+G+X:Subset5, GTR+I+G+X:Subset6, GTR+I+G+X:Subset7, HKY+I+G+X:Subset8, HKY+G+X:Subset9, TRN+G+X:Subset10, JC+I:Subset11, GTR+G+X:Subset12;

end;

**Appendix 5**

Best scoring IQ-TREE ML tree (SH-aLRT / UFboot at nodes)

Results of phylogenetic analysis

We recover Laccophilinae as sister to Cybistrinae+Copelatinae, the latter relationship being moderately supported (SH-aLRT=93.1/UFBoot=86). Within a monophyletic Copelatinae, we recover two main clades. The first clade comprises the genera *Agaporomorphus*, *Aglymbus*, *Copelatus*, *Lacconectus* and *Madaglymbus* (SH-aLRT=93.4/ UFBoot 57). The second clade comprises the genera *Capelatus*, *Exocelina* and *Liopterus* (SH-aLRT=88.4/ UFBoot 50). We infer *Exocelina* as monophyletic (SH-aLRT=99.6/UFBoot=99) and sister to the clade *Capelatus*+*Liopterus* (SH-aLRT=57/ UFBoot=27). Within *Exocelina*, we recover two main clades. The first one (SH-aLRT=31.8/ UFBoot=59) comprises Australian and New Caledonian species as well as the only lentic New Guinean *Exocelina* species *E. baliem*. In the second clade (SH-aLRT=16/ UFBoot=57), we recover the rest of Australian and New Caledonian species as well as the Hawaiian species *E. parvula*, the Chinese species *E. shizong*, the Vanuatu species *E. cheesmanae*, and the New Guinean *Exocelina* radiation. The latter is recovered as sister to the Australian underground aquifer species *E. abdita* as well as the epigean *E.* *elongatula* (SH-aLRT=74.7/ UFBoot=69).

General discussion of tree topology

The subfamilial relationships are moderately supported and the relationship between Cybistrinae and Laccophilinae is inconsistent with the most recent and comprehensive treatment of diving beetle phylogenetics [1]. Considering the lower genetic completeness and coverage of our dataset for these outgroups, we suggest that the phylogeny of [1] is more likely to represent valid subfamilial phylogenetic relationships than the ones inferred in this study. The phylogenetic inference for the ingroup is largely consistent with previous estimates based on overlapping molecular datasets [1-5]. The main discrepancy remains the placement of the South African genus *Capelatus* (one species) and Palearctic genus *Liopterus* (two species), recovered in some studies as sister to the rest of genera in Copelatinae except for *Exocelina* [2], or as sister to *Exocelina* [1, 3-5]. The latter relationship appears to be the most likely placement for these two genera and is partly supported by larval morphology and chaetotaxy [6].

References

1. Désamore A, Laenen B, Miller KB, Bergsten J: **Early burst in body size evolution is uncoupled from species diversification in diving beetles (Dytiscidae)**. *Molecular Ecology* 2018, **27**(4):979–993.

2. Balke M, Ribera I, Vogler AP: **MtDNA phylogeny and biogeography of Copelatinae, a highly diverse group of tropical diving beetles (Dytiscidae)**. *Molecular Phylogenetics and Evolution* 2004, **32**(3):866–880.

3. Bilton DT, Toussaint EFA, Turner CR, Balke M: **Capelatus prykeigen. et sp.n. (Coleoptera: Dytiscidae: Copelatinae) - a phylogenetically isolated diving beetle from the Western Cape of South Africa**. *Systematic Entomology* 2015, **40**(3):520–531.

4. Ribera I, Vogler AP, Balke M: **Phylogeny and diversification of diving beetles (Coleoptera: Dytiscidae)**. *Cladistics* 2008, **24**(4):563–590.

5. Toussaint EF, Hall R, Monaghan MT, Sagata K, Ibalim S, Shaverdo HV, Vogler AP, Pons J, Balke M: **The towering orogeny of New Guinea as a trigger for arthropod megadiversity**. *Nature Communications* 2014, **5**:4001.

6. Michat MC, Alarie Y, Miller KB: **Higher‐level phylogeny of diving beetles (C oleoptera: D ytiscidae) based on larval characters**. *Systematic Entomology* 2017, **42**(4):734–767.

**Appendix 6**

Best scoring IQ-TREE ML tree in newick format

(((Africophilus_sp.:0.4154829989,(Australphilus_montanus:0.2032891293,((Neptosternus_hydaticoides:0.1078091162,Philaccolilus_bellissimus:0.3984783206)95.3/93:0.0928276393,Philaccolus_sp.:0.2561216620)45.4/43:0.0488605991)43/28:0.0311618252)38.4/46:0.0152871947,Laccophilus_pictus:0.4343146547)88.7/64:0.0805884114,((Agabetes_acuductus:0.1789803698,((Austrodytes_plateni:0.2093753206,Cybister_tripunctatus:0.1925185164)44.9/57:0.0541371965,(Onychohydrus_scutellaris:0.1561902408,Spencerhydrus_latecinctus:0.1911124785)21.7/50:0.0477273145)89/48:0.0261721741)77.1/80:0.0311600395,((((Agaporomorphus_knischi_Peru_MB0751:0.2870248176,(Madaglymbus_ruthwildae_Madagascar_MB1244:0.0812661173,Madaglymbus_sp_Madagascar_MB0009:0.0730382987)100/100:0.1564089513)74.7/91:0.0702023542,(Aglymbus_leprieurii_French_Guyana_MB0307:0.0851047706,Aglymbus_pilatus_Venezuela_MB0645:0.0726020006)100/100:0.1629730191)18.7/59:0.0383390704,(((((Copelatus_antoniorum_MB4037_Oman_Batinah:0.1633702196,(Copelatus_distinguendens_Rodrigues_Island_MB3100:0.0960412324,(Copelatus_marginatus_New_Caledonia_MB3565:0.1747428865,Copelatus_tenebrosus_Sulawesi_MB4862:0.1015306188)71.1/53:0.0343774129)42.6/48:0.0420475988)96.9/90:0.0600812875,((Copelatus_assimilis_Gabon_MB3432:0.0377169882,Copelatus_gardineri_Seychelles_MB3182:0.0360531697)99.7/100:0.0968605581,Copelatus_bromeliarum_Trinidad_and_Tobago_MB1250:0.2958107362)85.2/57:0.0606423192)79.2/37:0.0398602610,Copelatus_glyphicus_MB4287_USA_California:0.2735950609)86.6/45:0.0387682277,Copelatus_daemeli_Australia_MB1607:0.1979379316)93.8/66:0.0694112897,((Lacconectus_atlas_Laos_MB5602:0.0284707859,Lacconectus_punctipennis_Borneo_MB0043:0.0345865792)97.1/99:0.0747146988,Lacconectus_sabahensis_Malaysia_MB0626:0.1664047472)99.9/100:0.1305277478)96.6/46:0.0676550079)93.4/57:0.0558411496,((Capelatus_prykei_South_Africa_MB3919:0.2053588412,(Liopterus_haemorrhoidalis_Germany_MB0249:0.0628416182,Liopterus_atriceps_Italy_MB0047:0.0165589482)100/100:0.2965055723)57/27:0.0167337521,(((((((((((((Exocelina_adelbert_MB1297_PNG_Madang:0.0051974406,(Exocelina_bewani_MB1296_PNG_Sandaun:0.0009051555,Exocelina_eggshaped_MB6979_Indonesia_Papua_WANO:0.0315391937)73.7/32:0.0023609966)54/86:0.0099407199,Exocelina_cyclops_MB3330_Indonesia_Papua:0.0199411352)99.6/99:0.0143651595,((((Exocelina_ibalimi_MB0657_PNG_Sandaun:0.0023050801,Exocelina_near_fume_MB6405_Indonesia_Papua:0.0052387581)97.8/99:0.0118624905,Exocelina_casuarina_MB6408_Indonesia_Papua:0.0147235794)67.7/71:0.0033521063,((Exocelina_keki_MB1530_PNG_Madang:0.0014317183,Exocelina_near_fume_MB4169_PNG_Madang:0.0042649429)100/100:0.0228227119,Exocelina_piusi_MB4921_PNG_EastSepik:0.0066648073)94.4/96:0.0113626790)99/99:0.0118771595,Exocelina_koroba_MB1292_PNG_SouthernHighlands:0.0313891790)97.5/94:0.0071826549)98.1/98:0.0087040679,Exocelina_mekilensis_MB0686_PNG_Sandaun:0.0324506327)89.3/87:0.0066591453,(Exocelina_menyamya_MB1377_PNG_Morobe:0.0142671245,Exocelina_pusilla_MB1364_PNG_Madang:0.0115151605)99.7/100:0.0133007961)70.2/80:0.0046121527,((((Exocelina_ambua_MB1290_PNG_SouthernHighlands:0.0033364857,(Exocelina_desii_MB1399_PNG_EHP:0.0010328249,Exocelina_simbaijimi_MB3312_PNG_WesternHighlands:0.0016606916)89.1/76:0.0010462502)95.3/98:0.0043514675,Exocelina_mendiensis_MB1337_PNG_SouthernHighlands:0.0047243419)98.1/99:0.0056495153,Exocelina_kumulensis_MB1360_PNG_Enga:0.0096602308)100/100:0.0131518325,(((Exocelina_inengensis_MB3309_PNG_WesternHighlands:0.0004354846,Exocelina_simbaiensis_MB3315_PNG_WesternHighlands:0.0000021088)100/100:0.0220184001,Exocelina_yoginofi_MB1302_PNG_EHP:0.0109135819)78.8/87:0.0042722292,(((((Exocelina_ketembang_MB0680_PNG_Sandaun:0.0026148060,Exocelina_may_MB0671_PNG_Sandaun:0.0023736427)89.6/77:0.0013372417,Exocelina_talaki_MB6415_Indonesia_Papua:0.0046754388)29.4/56:0.0007789754,Exocelina_wamena_MB7002_Indonesia_Papua:0.0048198968)91.2/63:0.0035636017,Exocelina_me_MB6411_Indonesia_Papua:0.0097474132)88/92:0.0028463580,((Exocelina_spcernyi_MB0066_Indonesia_Papua:0.0000021808,(Exocelina_aipo_MB6402_Papua:0.0000026225,Exocelina_manfredi_MB6414_Papua:0.0011065860)90.7/96:0.0040768074)73.5/67:0.0013368070,Exocelina_karmurensis_MB6406_Indonesia_Papua:0.0021161731)93.8/95:0.0042241981)99.2/100:0.0081745119)99.3/98:0.0059659598)91.2/95:0.0040117720)88.8/83:0.0033641514,(Exocelina_kinibeli_MB1379_PNG_Morobe:0.0050323382,Exocelina_ullrichi_MB1253_PNG_EHP:0.0126566128)100/100:0.0249110360)94.2/90:0.0043826423,(((((Exocelina_aipomek_MB3726_PNG_Sandaun:0.0020315292,Exocelina_sanctimontis_MB6412_Indonesia_Papua:0.0028877783)100/100:0.0476440812,Exocelina_erteldi_MB6404_Indonesia_Papua:0.0356248795)77.6/76:0.0082062913,(((((Exocelina_andakombiensis_MB1361_PNG_Gulf:0.0178900868,(((Exocelina_broschii_MB1300_PNG_Madang:0.0048477135,Exocelina_marinae_MB1291_PNG_SouthernHighlands:0.0000026415)81.4/64:0.0012366277,(Exocelina_mondmillensis_MB1525_PNG_Enga:0.0009841217,Exocelina_pseudomarinae_MB1287_PNG_Hela:0.0009776324)38.5/67:0.0014779101)57.7/59:0.0012757131,Exocelina_hintelmannae_MB1367_PNG_Gulf:0.0028063861)99.7/100:0.0080177538)98.6/97:0.0071494906,(((((Exocelina_damantiensis_MB1315_PNG_Morobe:0.0022255540,Exocelina_danae_MB0673_PNG_Sandaun:0.0008047668)50.9/68:0.0011076716,Exocelina_garaina_MB3876_PNG_Morobe:0.0013821601)85.7/94:0.0029410263,Exocelina_miriae_MB1374_PNG_Morobe:0.0137678865)84.8/72:0.0016256260,(Exocelina_injiensis_MB1376_PNG_Morobe:0.0063440984,Exocelina_kabwumensis_MB1285_PNG_Morobe:0.0060672488)71.7/57:0.0004348748)99.9/100:0.0090373679,(Exocelina_monae_MB1381_PNG_Morobe:0.0030569047,Exocelina_pseudomonae_MB1375_PNG_Morobe:0.0016940519)99.5/100:0.0085993267)93/94:0.0047571003)91.9/74:0.0036665214,(((Exocelina_jaseminae_MB1382_PNG_Morobe:0.0096213771,Exocelina_kailaki_MB3409_PNG_Central:0.0200115689)97.1/82:0.0087228302,((Exocelina_varirata_MB3303_PNG_NCD:0.0013777640,Exocelina_wareaga_MB3404_PNG_Central:0.0100950354)96.1/97:0.0115967759,Exocelina_woitapensis_MB3399_PNG_Central:0.0123584403)50.7/45:0.0061182436)67.7/53:0.0049237154,(Exocelina_marawaka_MB1366_PNG_EHP:0.0056647434,Exocelina_posmani_MB3406_PNG_Central:0.0040068173)99.9/100:0.0124368076)92.5/61:0.0045304783)60/24:0.0026789793,(Exocelina_oh_MB0261_PNG:0.0185254307,Exocelina_near_bacchusi_MB7001_Indonesia_Papua:0.0212557026)98.9/100:0.0123677469)85.6/69:0.0032570695,((((Exocelina_bacchusi_MB1521_PNG_EHP:0.0025357105,Exocelina_waraserensis_MB0257_PNG:0.0079758917)75.5/39:0.0005441235,Exocelina_yus_MB6531_PNG_PNG_MorobeHuon:0.0032210484)0/15:0.0000024702,Exocelina_herzogensis_MB1383_PNG_Morobe:0.0024066804)98.6/99:0.0080542753,Exocelina_pulchella_MB3408_PNG_Central:0.0165590061)99.5/82:0.0141841257)60.2/38:0.0030185790)94.3/81:0.0058912361,(((((Exocelina_haiaensis_MB0265_PNG:0.0000023882,Exocelina_tapiniensis_MB3306_PNG_Central:0.0006440869)99.6/100:0.0153299365,Exocelina_larsoni_MB1299_PNG_Madang:0.0166226366)66.1/41:0.0026081947,Exocelina_kokoda_MB3405_PNG_Central:0.0187579922)95.7/96:0.0094132993,Exocelina_mianminensis_MB0688_PNG_Sandaun:0.0456142716)93.6/44:0.0058966443,(Exocelina_morobensis_MB1313_PNG_Morobe:0.0029710171,Exocelina_sosanikai_MB3840_PNG_Morobe:0.0019787452)100/100:0.0249467487)99.7/99:0.0139135459)95.9/97:0.0048898200,(((((((((Exocelina_anggiensis_MB1272_Indonesia_WPapua:0.0056249706,Exocelina_arfakensis_MB1268_Indonesia_WPapua:0.0074273514)43.3/61:0.0042983385,Exocelina_polita_MB7004_Indonesia_WPapua:0.0072228704)96.5/94:0.0088869883,((Exocelina_evelyncheesmanae_MB1276_Indonesia_Papua:0.0037332407,Exocelina_manokwariensis_nokensis_MB1275_Indonesia_Papua:0.0028963631)94.1/98:0.0041829601,(Exocelina_manokwariensis_hendrichi_MB1321_Indonesia_WPapuaFakFak:0.0050369539,((Exocelina_testega_MB7009_Indonesia_WPapua:0.0043708877,Exocelina_manokwariensis_batanta_MB1281_Indonesia_WPapua:0.0078361370)93/94:0.0061389856,(Exocelina_alexanderi_MB7006_Indonesia_WPapua:0.0034353857,Exocelina_manokwariensis_MB6190_Indonesia_WPapua:0.0015828489)71.4/84:0.0072635893)82.3/73:0.0039387013)92.5/96:0.0045835745)98.7/99:0.0071877776)98.3/97:0.0066779126,((Exocelina_irianensis_MB4907_Indonesia_Papua_Nabire:0.0051084449,Exocelina_wondiwoensis_MB0056_Indonesia_WPapua:0.0038593775)92.3/98:0.0089734324,Exocelina_near_irianensis_MB6520_Indonesia_Papua_WANO:0.0220638385)89.7/93:0.0086489571)88.6/83:0.0023224693,((((((((((Exocelina_bewaniensis_MB1295_PNG_Sandaun:0.0041408237,(((Exocelina_kakapupu_MB4912_Indonesia_Papua:0.0038343443,(Exocelina_soppi_MB4911_Indonesia_Papua:0.0013502789,Exocelina_weylandensis_MB4908_Indonesia_Papua:0.0031653869)94.3/87:0.0022382803)1.1/64:0.0007399844,Exocelina_oceai_MB6504_Indonesia_Papua_WANO:0.0041596668)93.4/74:0.0029393296,(Exocelina_utowaensis_MB4905_Indonesia_Papua:0.0071778472,Exocelina_near_bewaniensis_MB6527_Indonesia_Papua_WANO:0.0020807938)44.7/31:0.0018092094)70.1/17:0.0003541874)84.4/36:0.0007573104,Exocelina_unipo_MB4903_Indonesia_Papua:0.0091567884)75.3/34:0.0007555812,Exocelina_cfutowaensis_MB7281_Papua_Waaf_FOJA:0.0009584731)75.4/67:0.0007270315,(Exocelina_pinocchio_MB3321_PNG_Madang:0.0186625652,Exocelina_wannangensis_MB3761_PNG_Madang:0.0016393194)98.1/100:0.0046798767)74.8/79:0.0016699399,Exocelina_mantembu_MB0060_Indonesia:0.0061371099)85.7/76:0.0014886850,(Exocelina_brahminensis_MB1298_PNG_Madang:0.0132760725,(Exocelina_pseudosoppi_MB4916_Indonesia_Papua_Nabire:0.0032482368,(Exocelina_cfbrahminensis_MB7287_Papua_FOJA1700m:0.0047902986,Exocelina_cfpseudosoppi_MB7286_Papua_FOJA1700m:0.0028299162)82.1/94:0.0034126704)98.4/97:0.0080112376)5/21:0.0003111238)63.8/61:0.0012348362,((Exocelina_pseudobifida_MB0659_PNG_Sandaun:0.0000021301,Exocelina_bifida_MB3722:0.0020141971)94.6/92:0.0073621280,Exocelina_foja_EWSPECIES_MB7282_Papua_Waaf_FOJA:0.0091339680)80.4/45:0.0037397858)49/28:0.0010862754,Exocelina_lembena_MB4922_PNG_EastSepik:0.0101854806)90.3/86:0.0062650983,Exocelina_pseudoeme_MB3759_PNG_Sandaun:0.0163502142)96.6/95:0.0069183748,((Exocelina_Brazza_MB6991_Indonesia_Papua:0.0139424167,Exocelina_oksibilensis_MB6997_Indonesia_Papua:0.0266195925)84.5/89:0.0038665776,(Exocelina_okbapensis_MB6993_Indonesia_Papua:0.0163640889,Exocelina_sp._MBXXXX_Papua_Tsinga:0.0129174927)89.9/91:0.0072999262)15.4/19:0.0008722430)79.4/70:0.0015036093)98.2/99:0.0056470042,(((((Exocelina_bismarckensis_MB1306_PNG_EHP:0.0011650096,Exocelina_gorokaensis_MB1307_PNG_EHP:0.0015446185)97/100:0.0033941548,(Exocelina_pseudoedeltraudae_MB1288_PNG_SouthernHighlands:0.0056782550,Exocelina_sandaunensis_MB0681_PNG_Sandaun:0.0028717399)92.4/96:0.0038105959)46.8/58:0.0015578224,(Exocelina_vovai_MB1372_PNG_Morobe:0.0015553664,Exocelina_herowana_MB6181_PNG_Simbu_EHP:0.0018483721)97.1/100:0.0053508144)82.3/91:0.0040790830,((Exocelina_bundiensis_MB1398_PNG_EHP:0.0043025548,((Exocelina_edeltraudae_MB1341_PNG_WesternHighlands:0.0028091404,(Exocelina_jimiensis_MB3311_PNG_WesternHighlands:0.0076012061,Exocelina_tariensis_MB1289_PNG_SHL_Hela:0.0041679982)76/78:0.0004914853)84.5/76:0.0010655782,Exocelina_sp._MB1369_PNG_Gulf:0.0028967382)82.2/72:0.0010929083)69.2/41:0.0003953733,Exocelina_michaelensis_MB4082_PNG_EHP:0.0010405515)93.1/98:0.0040543009)97.9/98:0.0065184732,((Exocelina_kisli_MB1373_PNG_:0.0046104528,Exocelina_knoepchen_MB1303_PNG_EHP:0.0032378378)96.2/100:0.0101714968,Exocelina_craterensis_MB6182_PNG_EHP:0.0219439552)51.8/65:0.0037176696)98/96:0.0055663589)94.2/96:0.0029669202,(((Exocelina_astrophallus_MB1529_PNG_Madang:0.0024840824,(Exocelina_atowaso_MB0267_PNG_Madang:0.0009277505,Exocelina_tabubilensis_MB7236_PNG:0.0125591452)69.8/70:0.0021231804)80.7/63:0.0032615428,Exocelina_pseudoastrophallus_MB6184_PNG_Lembena:0.0000027344)95.8/97:0.0179408912,Exocelina_munaso_MB0255_PNG:0.0477568963)94.1/99:0.0079627071)97.7/100:0.0054548995,Exocelina_skalei_MB4427_Indonesia_Papua:0.0396851014)86.2/71:0.0026268129,((Exocelina_bagus_MB4915_Indonesia_Papua:0.0353996436,(((((Exocelina_iratoispn_MB6985_Indonesia_Papua:0.0374841007,Exocelina_pulukensis_MB6514_Indonesia_Papua_WANO:0.0015537420)98.4/93:0.0130765101,Exocelina_wigodukensis_MB6530_Indonesia_Papua_Wano:0.0022677313)90.2/93:0.0042832020,(Exocelina_likui_MB6981_Indonesia_Papua_WANO:0.0076736396,Exocelina_pui_MB6518_Indonesia_Papua_WANO:0.0293694962)95.4/98:0.0078268432)92.3/92:0.0059559982,(Exocelina_tomhansi_MB6513_Indonesia_Papua_WANO:0.0017496110,Exocelina_ascendens_MB6409_Indonesia_Papua:0.0057135532)99.8/100:0.0217447704)73.1/83:0.0045401462,Exocelina_near_ransikiensis1_MB7235_Indonesia_Papua:0.0376618074)1.5/58:0.0027795215)94/96:0.0070939416,((Exocelina_ransikiensis_MB1269_Indonesia_Papua:0.0074968099,Exocelina_near_ransikiensis2_MB6185_Indonesia_WPapua:0.0058937904)93.1/94:0.0079955650,Exocelina_ransikiensis_MB6188_Indonesia_WPapua:0.0108057297)97/100:0.0139693289)70.5/48:0.0010422157)96.3/94:0.0045339866)83.9/87:0.0044069032)100/100:0.0645330329,(Exocelina_elongatula_MB1850_Australia_NSW:0.0988935055,Exocelina_abdita_MB0297_Australia_NT:0.1408263192)46.7/82:0.0251961782)74.7/69:0.0127696107,(((((Exocelina_aubeii_MB0693_NewCaledonia:0.0197302529,Exocelina_cheesmanae_MB1396_Vanuatu_EspirituSanto:0.0199918527)100/100:0.0619343654,(((((((Exocelina_barbarae_MB0121_NewCaledonia_NProv:0.0011592877,Exocelina_bimaculata_MB0163_NewCaledonia_NProv:0.0063663692)92.2/88:0.0037054296,Exocelina_gelima_MB0403_NewCaledonia:0.0037800504)80.7/56:0.0043751334,Exocelina_poellabauerae_MB0036_NewCaledonia_NProv:0.0078859061)99.9/100:0.0171209561,Exocelina_nielsi_MB0166_NewCaledonia_SProv:0.0388966684)78.2/45:0.0057515644,Exocelina_remyi_MB0132_NewCaledonia_SProv:0.0292055277)87.9/42:0.0043243574,(Exocelina_brownei_MB0139_NewCaledonia_SProv:0.0330048710,(Exocelina_flammi_MB0170_NewCaledonia_NProv:0.0054243245,Exocelina_perfecta_MB0168_NewCaledonia_NProv:0.0041989257)100/100:0.0261074947)74.1/40:0.0022254065)99.9/100:0.0202160014,(((Exocelina_brunoi_MB0019_NewCaledonia_SProv:0.0241656469,Exocelina_staneki_MB0413_NewCaledonia:0.0280882220)42.5/78:0.0026962388,(Exocelina_charlottae_MB0254_NewCaledonia_SProv:0.0259409618,((Exocelina_interrupta_MB0253_NewCaledonia_SProv:0.0067358513,Exocelina_subjecta_MB0130_NewCaledonia_SProv:0.0562917375)97.4/100:0.0067916743,Exocelina_rotteri_MB0135_NewCaledonia_SProv:0.0092215018)98.3/100:0.0097673190)66/64:0.0054204867)98/99:0.0100334866,(((Exocelina_burwelli_MB0409_NewCaledonia:0.0083673531,(Exocelina_niklasi_MB0039_NewCaledonia_NProv:0.0054393618,Exocelina_gaulorum_MB0123_NewCaledonia_NProv:0.0024215471)100/100:0.0173687555)27.5/43:0.0041691065,Exocelina_simoni_MB0020_NewCaledonia_SProv:0.0137312611)28.7/75:0.0071749267,Exocelina_commatifera_MB0090_NewCaledonia_NProv:0.0233713402)99.7/100:0.0177089025)95.4/92:0.0107601476)99.7/99:0.0157996950)12.6/9:0.0037852390,(Exocelina_creuxorum_MB0406_NewCaledonia:0.0293082520,(Exocelina_feryi_MB0142_NewCaledonia_SProv:0.0000025061,Exocelina_leae_MB0037_NewCaledonia_NProv:0.0053083715)99.1/100:0.0340558647)100/100:0.0545803114)94.1/67:0.0079373103,Exocelina_shizong_MB0050_China:0.0755587439)48.2/37:0.0040968093,Exocelina_parvula_MB1261_USA_Hawaii_KauaiIsland:0.0828268180)98/89:0.0151312875)89.3/81:0.0140901110,Exocelina_australis_MB0296_Australia:0.1095453730)86/68:0.0150731863,(Exocelina_ater_MB2669_Australia_WesternAustralia:0.0222509864,Exocelina_simplex_MB1445_Australia_SA:0.0427955022)100/100:0.0654425752)16/57:0.0059971379,(((((((Exocelina_australiae_MB1932_Australia_NSW:0.0045783897,Exocelina_sp._MB1503_Australia_SouthAustralia:0.0097354564)82.2/45:0.0043436513,Exocelina_sp._MB0295_Australia_Tasmania:0.0064204033)99.8/100:0.0170146866,(Exocelina_melanaria_MB1911_Australia_QLD:0.0125213277,Exocelina_sp._MB2521_Australia_QLD:0.0268906027)35.4/59:0.0029041192)80.5/82:0.0039033932,Exocelina_sp._MB1759_Australia_QLD:0.0231801621)100/100:0.0745763139,(((Exocelina_boulevardi_MB1818_Australia_NSW:0.0261389734,Exocelina_sp._MB1717_Australia_Tasmania:0.0477935159)82.5/79:0.0097552862,Exocelina_sp._MB0107_Australia:0.0460142770)100/100:0.0299962769,(((Exocelina_gapa_MB1813_Australia_QLD:0.0430949116,Exocelina_sp._MB1882_Australia_QLD:0.0488370884)97.4/98:0.0210130626,((Exocelina_sp._MB1747_Australia_QLD:0.0348556562,((Exocelina_sp._MB1770_Australia_QLD:0.0098329336,(Exocelina_sp._MB1788_Australia_NSW:0.0084663060,Exocelina_sp._MB1836_Australia_NSW:0.0024732197)94.1/58:0.0039059532)13.2/27:0.0013792612,(Exocelina_sp._MB1881_Australia_QLD:0.0000028683,Exocelina_rasilis_MB0086_Australia_QLD:0.0000934231)99.9/66:0.0137066669)98.9/100:0.0294465804)78.9/46:0.0148624738,Exocelina_sp._MB1910_Australia_QLD:0.0525001538)98.5/100:0.0196660456)95.9/92:0.0195776875,Exocelina_sp._MB1903_Australia_QLD:0.1304255836)93.5/95:0.0155480180)94.2/96:0.0104055854)98.9/98:0.0181259217,(((Exocelina_ferruginea_MB1501_Australia_SouthAustralia:0.0214982144,((Exocelina_sp._MB1710_Australia_WesternAustralia:0.0077600735,Exocelina_cfferruginea_MB7175_Australia_Qld:0.0066863847)96.6/99:0.0216065273,Exocelina_baliem_MB7011_Indonesia_Papua:0.0057110201)79.6/83:0.0067955336)30.6/24:0.0015626720,Exocelina_sp._MB0282_Australia_WA:0.0329878861)95.9/83:0.0096794932,(Exocelina_punctipennis_MB1804_Australia_NSW:0.0054481542,Exocelina_inexpectata_MB7520_NewCaledonia_NProv:0.0168878484)94.3/98:0.0170596256)100/100:0.0601910718)98.2/95:0.0140421653,(((Exocelina_maculata_MB0415_NewCaledonia:0.0352326342,(Exocelina_novaecaledoniae_MB0137_NewCaledonia_SProv:0.0031536116,Exocelina_ouin_MB0410_NewCaledonia:0.0014952900)99.9/100:0.0311105123)99.1/100:0.0255150931,Exocelina_monteithi_MB0292_NewCaledonia_NProv:0.0758178114)99.4/100:0.0251941422,Exocelina_sp._MB2536_Australia_NSW:0.0840617237)99.7/93:0.0278784117)31.8/59:0.0062494699)99.6/99:0.1468666053)88.4/50:0.0592104247)94.3/84:0.1226978378)93.1/86:0.0851503186,Sandracottus_bakewellii:0.4355113810);

**Appendix 7**

Best BEAST analysis chronogram with median ages

**Appendix 8**

Best BEAST analysis chronogram with median ages in newick format

(Sandracottus_bakewellii:127.11239577992781,(((Agabetes_acuductus:65.54550240876071,((Austrodytes_plateni:42.85136397338296,Cybister_tripunctatus:42.85136397338296):11.422991844835408,(Onychohydrus_scutellaris:35.45378552411789,Spencerhydrus_latecinctus:35.45378552411789):18.820570294100477):11.271146590542344):36.78034771491092,(Laccophilus_pictus:82.09683323145487,('Africophilus_sp.':75.54969368384455,(Australphilus_montanus:65.75508208954552,('Philaccolus_sp.':54.70075325376091,(Neptosternus_hydaticoides:35.242238191775755,Philaccolilus_bellissimus:35.242238191775755):19.458515061985153):11.054328835784617):9.794611594299028):6.547139547610314):20.229016892216762):9.764528416384437,((((Aglymbus_leprieurii_French_Guyana_MB0307:27.14599809029417,Aglymbus_pilatus_Venezuela_MB0645:27.14599809029417):40.77318267364048,(Agaporomorphus_knischi_Peru_MB0751:54.26056143734134,(Madaglymbus_ruthwildae_Madagascar_MB1244:20.328702889092796,Madaglymbus_sp_Madagascar_MB0009:20.328702889092796):33.93185854824854):13.65861932659331):10.371257877478058,((Lacconectus_sabahensis_Malaysia_MB0626:35.74665341771302,(Lacconectus_atlas_Laos_MB5602:8.514499484403458,Lacconectus_punctipennis_Borneo_MB0043:8.514499484403458):27.23215393330956):31.290134893161095,(Copelatus_daemeli_Australia_MB1607:56.57431309594173,(Copelatus_glyphicus_MB4287_USA_California:49.31902326533083,((Copelatus_bromeliarum_Trinidad_and_Tobago_MB1250:34.95043163552718,(Copelatus_assimilis_Gabon_MB3432:10.332391428494887,Copelatus_gardineri_Seychelles_MB3182:10.332391428494887):24.618040207032294):10.423284203888585,(Copelatus_antoniorum_MB4037_Oman_Batinah:33.953249472255266,(Copelatus_distinguendens_Rodrigues_Island_MB3100:28.6451914577966,(Copelatus_marginatus_New_Caledonia_MB3565:22.420461974539634,Copelatus_tenebrosus_Sulawesi_MB4862:22.420461974539634):6.224729483256937):5.308058014458695):11.420466367160472):3.945307425915061):7.255289830610906):10.462475214932383):11.25365033053859):14.33933687429662,((Capelatus_prykei_South_Africa_MB3919:66.55430136071497,(Liopterus_haemorrhoidalis_Germany_MB0249:11.451115985781556,Liopterus_atriceps_Italy_MB0047:11.451115985781556):55.10318537493342):11.046475666583575,((('Exocelina_sp._MB2536_Australia_NSW':31.418147680970904,(Exocelina_monteithi_MB0292_NewCaledonia_NProv:22.278780809745953,(Exocelina_maculata_MB0415_NewCaledonia:11.480750302172623,(Exocelina_novaecaledoniae_MB0137_NewCaledonia_SProv:1.1347574234255262,Exocelina_ouin_MB0410_NewCaledonia:1.1347574234255262):10.345992878747097):10.79803050757333):9.13936687122495):11.951003240646429,(((Exocelina_punctipennis_MB1804_Australia_NSW:4.202154257845862,Exocelina_inexpectata_MB7520_NewCaledonia_NProv:4.202154257845862):8.70023391446287,('Exocelina_sp._MB0282_Australia_WA':10.02373427537296,(Exocelina_ferruginea_MB1501_Australia_SouthAustralia:9.177735744538623,(Exocelina_baliem_MB7011_Indonesia_Papua:6.362471184674774,('Exocelina_sp._MB1710_Australia_WesternAustralia':2.497523867484148,Exocelina_cfferruginea_MB7175_Australia_Qld:2.497523867484148):3.8649473171906266):2.8152645598638486):0.8459985308343363):2.878653896935745):25.64029212615867,(('Exocelina_sp._MB1759_Australia_QLD':9.13433128789191,((Exocelina_melanaria_MB1911_Australia_QLD:5.679775399507591,'Exocelina_sp._MB2521_Australia_QLD':5.679775399507591):1.4938363874783676,('Exocelina_sp._MB0295_Australia_Tasmania':2.8103414302117216,(Exocelina_australiae_MB1932_Australia_NSW:1.6607074561251807,'Exocelina_sp._MB1503_Australia_SouthAustralia':1.6607074561251807):1.1496339740865409):4.363270356774208):1.9607195009059808):24.4600275633899,(('Exocelina_sp._MB0107_Australia':15.630456292215158,(Exocelina_boulevardi_MB1818_Australia_NSW:12.055379885394572,'Exocelina_sp._MB1717_Australia_Tasmania':12.055379885394572):3.5750764068205854):14.430131700951279,('Exocelina_sp._MB1903_Australia_QLD':25.698853435879442,((Exocelina_gapa_MB1813_Australia_QLD:13.774985199990496,'Exocelina_sp._MB1882_Australia_QLD':13.774985199990496):6.580589327391266,('Exocelina_sp._MB1910_Australia_QLD':14.336014381793248,('Exocelina_sp._MB1747_Australia_QLD':10.392338395942048,(('Exocelina_sp._MB1881_Australia_QLD':0.8442656096594732,Exocelina_rasilis_MB0086_Australia_QLD:0.8442656096594732):2.927000927961231,('Exocelina_sp._MB1770_Australia_QLD':2.8525054445517526,('Exocelina_sp._MB1788_Australia_NSW':1.3539838651573461,'Exocelina_sp._MB1836_Australia_NSW':1.3539838651573461):1.4985215793944064):0.9187610930689516):6.621071858321372):3.9436759858512005):6.019560145588514):5.34327890849768):4.361734557286994):3.533770858115375):4.948321447185563):4.826470623149959):2.6318265641692022,((Exocelina_ater_MB2669_Australia_WesternAustralia:11.775521709022186,Exocelina_simplex_MB1445_Australia_SA:11.775521709022186):31.340770621220585,(Exocelina_australis_MB0296_Australia:38.42710258912406,((Exocelina_parvula_MB1261_USA_Hawaii_KauaiIsland:29.077520922678133,(Exocelina_shizong_MB0050_China:27.55244033155877,((Exocelina_creuxorum_MB0406_NewCaledonia:9.552619534448283,(Exocelina_feryi_MB0142_NewCaledonia_SProv:1.1568683185813882,Exocelina_leae_MB0037_NewCaledonia_NProv:1.1568683185813882):8.395751215866923):15.801712374489327,((Exocelina_aubeii_MB0693_NewCaledonia:6.561238880969682,Exocelina_cheesmanae_MB1396_Vanuatu_EspirituSanto:6.561238880969682):16.813185799438173,(((Exocelina_brownei_MB0139_NewCaledonia_SProv:10.384827127027265,(Exocelina_flammi_MB0170_NewCaledonia_NProv:1.798118399475456,Exocelina_perfecta_MB0168_NewCaledonia_NProv:1.798118399475456):8.586708727551837):1.3057408137212008,(Exocelina_remyi_MB0132_NewCaledonia_SProv:9.92123935694326,(Exocelina_nielsi_MB0166_NewCaledonia_SProv:8.519307426581094,(Exocelina_poellabauerae_MB0036_NewCaledonia_NProv:3.1839877935780976,(Exocelina_gelima_MB0403_NewCaledonia:1.9104999832812695,(Exocelina_barbarae_MB0121_NewCaledonia_NProv:1.0829404175007795,Exocelina_bimaculata_MB0163_NewCaledonia_NProv:1.0829404175007795):0.82755956578049):1.2734878102968281):5.335319633002996):1.4019319303621671):1.7693285838052333):6.674004239633234,((Exocelina_commatifera_MB0090_NewCaledonia_NProv:8.419048073785419,(Exocelina_simoni_MB0020_NewCaledonia_SProv:5.844249170543435,(Exocelina_burwelli_MB0409_NewCaledonia:4.24644537153884,(Exocelina_niklasi_MB0039_NewCaledonia_NProv:0.9402758784985679,Exocelina_gaulorum_MB0123_NewCaledonia_NProv:0.9402758784985679):3.306169493040244):1.5978037990045948):2.574798903241984):6.066762193863241,((Exocelina_brunoi_MB0019_NewCaledonia_SProv:9.610398036999598,Exocelina_staneki_MB0413_NewCaledonia:9.610398036999598):1.7648473025896152,(Exocelina_charlottae_MB0254_NewCaledonia_SProv:9.727451263338523,(Exocelina_rotteri_MB0135_NewCaledonia_SProv:7.719404462914184,(Exocelina_interrupta_MB0253_NewCaledonia_SProv:6.168401608823615,Exocelina_subjecta_MB0130_NewCaledonia_SProv:6.168401608823615):1.5510028540905694):2.0080468004243386):1.6477940762507188):3.110564928059418):3.8787619127330686):5.009852500026156):1.979907228529754):2.1981084226211607):1.5250805911193623):5.539412182836699,((Exocelina_elongatula_MB1850_Australia_NSW:24.043086929673578,Exocelina_abdita_MB0297_Australia_NT:24.043086929673578):8.116423633259771,(((Exocelina_kinibeli_MB1379_PNG_Morobe:3.38497556518179,Exocelina_ullrichi_MB1253_PNG_EHP:3.38497556518179):11.674009037780948,(((Exocelina_menyamya_MB1377_PNG_Morobe:4.795163676323895,Exocelina_pusilla_MB1364_PNG_Madang:4.795163676323895):7.7313370062848605,(Exocelina_mekilensis_MB0686_PNG_Sandaun:11.123297342034974,((Exocelina_cyclops_MB3330_Indonesia_Papua:6.124150585986499,(Exocelina_adelbert_MB1297_PNG_Madang:3.4261721892492574,(Exocelina_bewani_MB1296_PNG_Sandaun:2.8290615793597595,Exocelina_eggshaped_MB6979_Indonesia_Papua_WANO:2.8290615793597595):0.5971106098894978):2.6979783967372413):3.499120038029858,(Exocelina_koroba_MB1292_PNG_SouthernHighlands:8.26589911315672,((Exocelina_casuarina_MB6408_Indonesia_Papua:4.8186014608785825,(Exocelina_ibalimi_MB0657_PNG_Sandaun:1.2168750121289946,Exocelina_near_fume_MB6405_Indonesia_Papua:1.2168750121289946):3.6017264487496163):1.443056554999913,(Exocelina_piusi_MB4921_PNG_EastSepik:3.7757632712189775,(Exocelina_keki_MB1530_PNG_Madang:0.9296431132368639,Exocelina_near_fume_MB4169_PNG_Madang:0.9296431132368639):2.8461201579821136):2.485894744659518):2.004241097278225):1.357371510859636):1.500026718018617):1.403203340573782):1.1244508627711696,((Exocelina_kumulensis_MB1360_PNG_Enga:4.4204408189506665,(Exocelina_mendiensis_MB1337_PNG_SouthernHighlands:2.2936007939141803,(Exocelina_ambua_MB1290_PNG_SouthernHighlands:1.1855894557804447,(Exocelina_desii_MB1399_PNG_EHP:0.5693363503345381,Exocelina_simbaijimi_MB3312_PNG_WesternHighlands:0.5693363503345381):0.6162531054459066):1.1080113381337355):2.126840025036458):5.968224253732302,((Exocelina_yoginofi_MB1302_PNG_EHP:5.231377101150315,(Exocelina_inengensis_MB3309_PNG_WesternHighlands:0.17521197217379836,Exocelina_simbaiensis_MB3315_PNG_WesternHighlands:0.17521197217379836):5.056165128976517):1.8211664566459405,((Exocelina_karmurensis_MB6406_Indonesia_Papua:1.3902752166148247,(Exocelina_spcernyi_MB0066_Indonesia_Papua:1.0554783460218573,(Exocelina_aipo_MB6402_Papua:0.3493454710101247,Exocelina_manfredi_MB6414_Papua:0.3493454710101247):0.7061328750117326):0.33479687059296737):2.2449090252408013,(Exocelina_me_MB6411_Indonesia_Papua:2.8160427512486024,(Exocelina_wamena_MB7002_Indonesia_Papua:1.8686296727510125,(Exocelina_talaki_MB6415_Indonesia_Papua:1.4850141066051492,(Exocelina_ketembang_MB0680_PNG_Sandaun:0.9708229717173538,Exocelina_may_MB0671_PNG_Sandaun:0.9708229717173538):0.5141911348877954):0.3836155661458349):0.9474130784976182):0.8191414906070236):3.4173593159406295):3.3361215148866847):3.262286472696985):1.4080330575828128):1.9980115585982219,((((Exocelina_morobensis_MB1313_PNG_Morobe:0.92340496231337,Exocelina_sosanikai_MB3840_PNG_Morobe:0.92340496231337):9.696906550836744,(Exocelina_mianminensis_MB0688_PNG_Sandaun:9.142984366219892,(Exocelina_kokoda_MB3405_PNG_Central:6.111631673980085,(Exocelina_larsoni_MB1299_PNG_Madang:4.822009852500699,(Exocelina_haiaensis_MB0265_PNG:0.2524061648169891,Exocelina_tapiniensis_MB3306_PNG_Central:0.2524061648169891):4.569603687683738):1.2896218214793862):3.031352692239807):1.4773271469302216):3.649749706250944,((Exocelina_erteldi_MB6404_Indonesia_Papua:9.260519511818927,(Exocelina_aipomek_MB3726_PNG_Sandaun:1.0148360688627633,Exocelina_sanctimontis_MB6412_Indonesia_Papua:1.0148360688627633):8.245683442956164):2.8039310991011632,((Exocelina_pulchella_MB3408_PNG_Central:5.820263877568436,(Exocelina_herzogensis_MB1383_PNG_Morobe:1.7492333647137315,(Exocelina_yus_MB6531_PNG_PNG_MorobeHuon:1.5628940762624524,(Exocelina_bacchusi_MB1521_PNG_EHP:1.3369690128023421,Exocelina_waraserensis_MB0257_PNG:1.3369690128023421):0.2259250634601102):0.18633928845127912):4.071030512854705):5.038640945684207,((Exocelina_oh_MB0261_PNG:5.7730126022581345,Exocelina_near_bacchusi_MB7001_Indonesia_Papua:5.7730126022581345):3.4754734543663375,(((Exocelina_marawaka_MB1366_PNG_EHP:2.1090411626943535,Exocelina_posmani_MB3406_PNG_Central:2.1090411626943535):4.863193217900815,((Exocelina_jaseminae_MB1382_PNG_Morobe:3.8230981894824367,Exocelina_kailaki_MB3409_PNG_Central:3.8230981894824367):1.919505611622327,(Exocelina_woitapensis_MB3399_PNG_Central:3.7032455292336124,(Exocelina_varirata_MB3303_PNG_NCD:1.5838917735892153,Exocelina_wareaga_MB3404_PNG_Central:1.5838917735892153):2.119353755644397):2.0393582718711514):1.229630579490376):1.2695872287072802,((Exocelina_andakombiensis_MB1361_PNG_Gulf:4.447905642115302,(Exocelina_hintelmannae_MB1367_PNG_Gulf:1.5483821933560193,((Exocelina_broschii_MB1300_PNG_Madang:0.8241219583942723,Exocelina_marinae_MB1291_PNG_SouthernHighlands:0.8241219583942723):0.17019580090814657,(Exocelina_mondmillensis_MB1525_PNG_Enga:0.42284909984829255,Exocelina_pseudomarinae_MB1287_PNG_Hela:0.42284909984829255):0.5714686594540979):0.5540644340536289):2.8995234487592825):2.502052060572325,((Exocelina_monae_MB1381_PNG_Morobe:0.9452997367984182,Exocelina_pseudomonae_MB1375_PNG_Morobe:0.9452997367984182):4.21145072509556,((Exocelina_injiensis_MB1376_PNG_Morobe:2.7000500192341406,Exocelina_kabwumensis_MB1285_PNG_Morobe:2.7000500192341406):0.37735339696607184,(Exocelina_miriae_MB1374_PNG_Morobe:2.4346808640394215,(Exocelina_garaina_MB3876_PNG_Morobe:0.9856268318099808,(Exocelina_damantiensis_MB1315_PNG_Morobe:0.5517395817952035,Exocelina_danae_MB0673_PNG_Sandaun:0.5517395817952035):0.43388725001477724):1.4490540322294407):0.6427225521607909):2.079347045693794):1.7932072407936488):1.2918639066147932):1.006664447322052):1.6104187666281717):1.2055457876674467):2.205610608480967):1.359354243726358,(((Exocelina_ransikiensis_MB6188_Indonesia_WPapua:5.507992599595184,(Exocelina_ransikiensis_MB1269_Indonesia_Papua:2.3024062208266116,Exocelina_near_ransikiensis2_MB6185_Indonesia_WPapua:2.3024062208266116):3.2055863787686008):7.994208182965352,(Exocelina_bagus_MB4915_Indonesia_Papua:11.227849478556195,(Exocelina_near_ransikiensis1_MB7235_Indonesia_Papua:9.783037002051444,((Exocelina_tomhansi_MB6513_Indonesia_Papua_WANO:1.5792688174275327,Exocelina_ascendens_MB6409_Indonesia_Papua:1.5792688174275327):6.94305717117328,((Exocelina_likui_MB6981_Indonesia_Papua_WANO:4.22582879312445,Exocelina_pui_MB6518_Indonesia_Papua_WANO:4.22582879312445):2.4300463586307472,(Exocelina_wigodukensis_MB6530_Indonesia_Papua_Wano:5.411066812867972,(Exocelina_iratoispn_MB6985_Indonesia_Papua:2.8754831075846994,Exocelina_pulukensis_MB6514_Indonesia_Papua_WANO:2.8754831075846994):2.535583705283244):1.2448083388872249):1.8664508368456438):1.2607110134506314):1.444812476504751):2.274351304004341):0.5253595531315369,(Exocelina_skalei_MB4427_Indonesia_Papua:13.096661648224,((Exocelina_munaso_MB0255_PNG:9.087627807355915,(Exocelina_pseudoastrophallus_MB6184_PNG_Lembena:2.7873310866475407,(Exocelina_astrophallus_MB1529_PNG_Madang:1.4869537829206934,(Exocelina_atowaso_MB0267_PNG_Madang:1.148391155364422,Exocelina_tabubilensis_MB7236_PNG:1.148391155364422):0.3385626275562714):1.3003773037268473):6.300296720708403):2.3027278972180767,(((Exocelina_craterensis_MB6182_PNG_EHP:5.23779142610708,(Exocelina_kisli_MB1373_PNG_:1.605965404846529,Exocelina_knoepchen_MB1303_PNG_EHP:1.605965404846529):3.631826021260551):2.052229397572347,(((Exocelina_vovai_MB1372_PNG_Morobe:0.75759085320243,Exocelina_herowana_MB6181_PNG_Simbu_EHP:0.75759085320243):2.077636259760368,((Exocelina_bismarckensis_MB1306_PNG_EHP:0.7287837906316668,Exocelina_gorokaensis_MB1307_PNG_EHP:0.7287837906316668):1.5889655420721596,(Exocelina_pseudoedeltraudae_MB1288_PNG_SouthernHighlands:1.469895650303755,Exocelina_sandaunensis_MB0681_PNG_Sandaun:1.469895650303755):0.8478536824000713):0.5174777802589716):1.321913739405204,(Exocelina_michaelensis_MB4082_PNG_EHP:2.8321994804771293,(Exocelina_bundiensis_MB1398_PNG_EHP:2.5386645927146674,('Exocelina_sp._MB1369_PNG_Gulf':2.077220987501235,(Exocelina_edeltraudae_MB1341_PNG_WesternHighlands:1.7321101129822978,(Exocelina_jimiensis_MB3311_PNG_WesternHighlands:1.4973980531784576,Exocelina_tariensis_MB1289_PNG_SHL_Hela:1.4973980531784576):0.23471205980384013):0.3451108745189373):0.4614436052134323):0.29353488776246195):1.3249413718908727):3.1328799713114535):3.0130421910133123,(((Exocelina_near_irianensis_MB6520_Indonesia_Papua_WANO:5.2443225688194985,(Exocelina_irianensis_MB4907_Indonesia_Papua_Nabire:2.4294795051560385,Exocelina_wondiwoensis_MB0056_Indonesia_WPapua:2.4294795051560385):2.81484306366346):2.785678616685601,((Exocelina_polita_MB7004_Indonesia_WPapua:3.3791544825776327,(Exocelina_anggiensis_MB1272_Indonesia_WPapua:2.238348709495142,Exocelina_arfakensis_MB1268_Indonesia_WPapua:2.238348709495142):1.1408057730825192):2.3386171016359327,((Exocelina_evelyncheesmanae_MB1276_Indonesia_Papua:1.5681153986093364,Exocelina_manokwariensis_nokensis_MB1275_Indonesia_Papua:1.5681153986093364):2.445285987195831,(Exocelina_manokwariensis_hendrichi_MB1321_Indonesia_WPapuaFakFak:3.1423380235841165,((Exocelina_testega_MB7009_Indonesia_WPapua:1.4071467445836277,Exocelina_manokwariensis_batanta_MB1281_Indonesia_WPapua:1.4071467445836277):1.1060905335163937,(Exocelina_alexanderi_MB7006_Indonesia_WPapua:0.8342935222754022,Exocelina_manokwariensis_MB6190_Indonesia_WPapua:0.8342935222754022):1.6789437558246192):0.6291007454840951):0.8710633622210509):1.7043701984084265):2.312229601291534):0.901143979472451,(((Exocelina_Brazza_MB6991_Indonesia_Papua:6.101929671823854,Exocelina_oksibilensis_MB6997_Indonesia_Papua:6.101929671823854):1.6967601094672773,(Exocelina_okbapensis_MB6993_Indonesia_Papua:4.032971029290394,'Exocelina_sp._MBXXXX_Papua_Tsinga':4.032971029290394):3.765718752000737):0.6186472039970994,(Exocelina_pseudoeme_MB3759_PNG_Sandaun:6.700991861590879,(Exocelina_lembena_MB4922_PNG_EastSepik:5.082289178188219,((Exocelina_foja_EWSPECIES_MB7282_Papua_Waaf_FOJA:2.578561254629534,(Exocelina_pseudobifida_MB0659_PNG_Sandaun:0.4515604341791857,Exocelina_bifida_MB3722:0.4515604341791857):2.1270008204503483):2.069469156243315,((Exocelina_brahminensis_MB1298_PNG_Madang:3.7589871495191574,(Exocelina_pseudosoppi_MB4916_Indonesia_Papua_Nabire:1.8801576803442686,(Exocelina_cfbrahminensis_MB7287_Papua_FOJA1700m:1.1241120388228865,Exocelina_cfpseudosoppi_MB7286_Papua_FOJA1700m:1.1241120388228865):0.7560456415213821):1.8788294691748888):0.38872279349459404,(Exocelina_mantembu_MB0060_Indonesia:3.809067299339162,((Exocelina_pinocchio_MB3321_PNG_Madang:2.5383725579909537,Exocelina_wannangensis_MB3761_PNG_Madang:2.5383725579909537):0.9137577078195989,(Exocelina_cfutowaensis_MB7281_Papua_Waaf_FOJA:3.097980425546808,(Exocelina_unipo_MB4903_Indonesia_Papua:2.8302540526442073,(Exocelina_bewaniensis_MB1295_PNG_Sandaun:2.48136250039137,((Exocelina_utowaensis_MB4905_Indonesia_Papua:1.348284438690314,Exocelina_near_bewaniensis_MB6527_Indonesia_Papua_WANO:1.348284438690314):0.9416276467250952,(Exocelina_oceai_MB6504_Indonesia_Papua_WANO:1.6572481624175452,(Exocelina_kakapupu_MB4912_Indonesia_Papua:1.4047735793616027,(Exocelina_soppi_MB4911_Indonesia_Papua:0.7308391348372538,Exocelina_weylandensis_MB4908_Indonesia_Papua:0.7308391348372538):0.6739344445243489):0.2524745830559425):0.6326639229978639):0.19145041497596083):0.3488915522528373):0.26772637290260093):0.3541498402637444):0.3569370335286379):0.3386426436745893):0.5003204678590691):0.4342587673153986):1.6187026834026597):1.7163451236973515):0.5138081796893204):1.3719178497151887):1.0872926898812523):1.7063059436500083):0.9308986874680727):1.6018551274353428):1.4275806984335446):15.102514401372389):2.457422542581483):3.8101694836092292):4.68918974111871):2.884685155543764):31.599799541512013):15.028998488410778):19.46060302434674):15.022017239871744);

**Appendix 9**

Distribution of groups resulting from geospatial analysis plotted on the map of New Guinea.


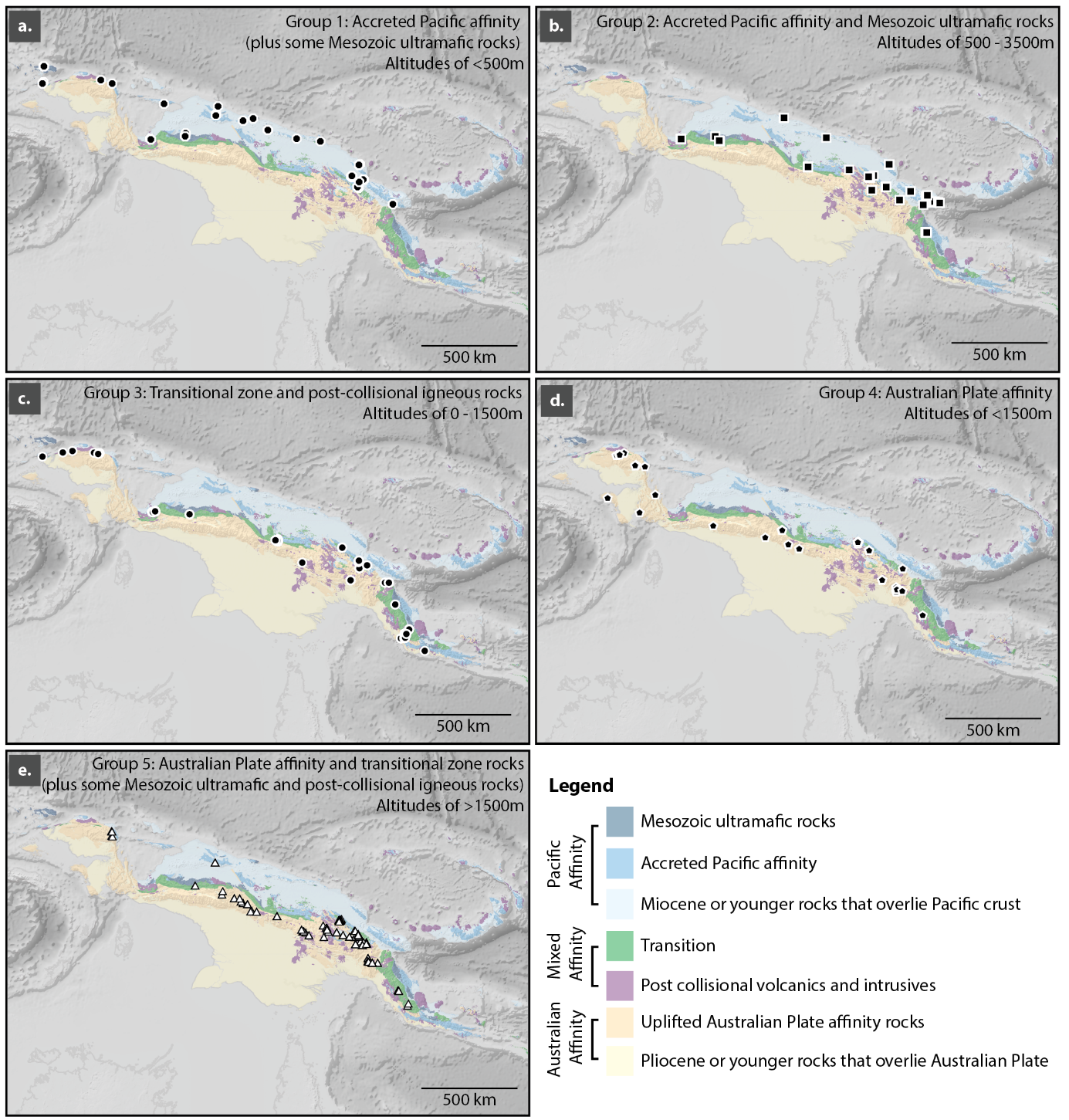


**Appendix 10**

2D histograms (“Heat maps”) for geology, altitude and geospatial analysis groups and projected onto the phylogenetic hypothesis.


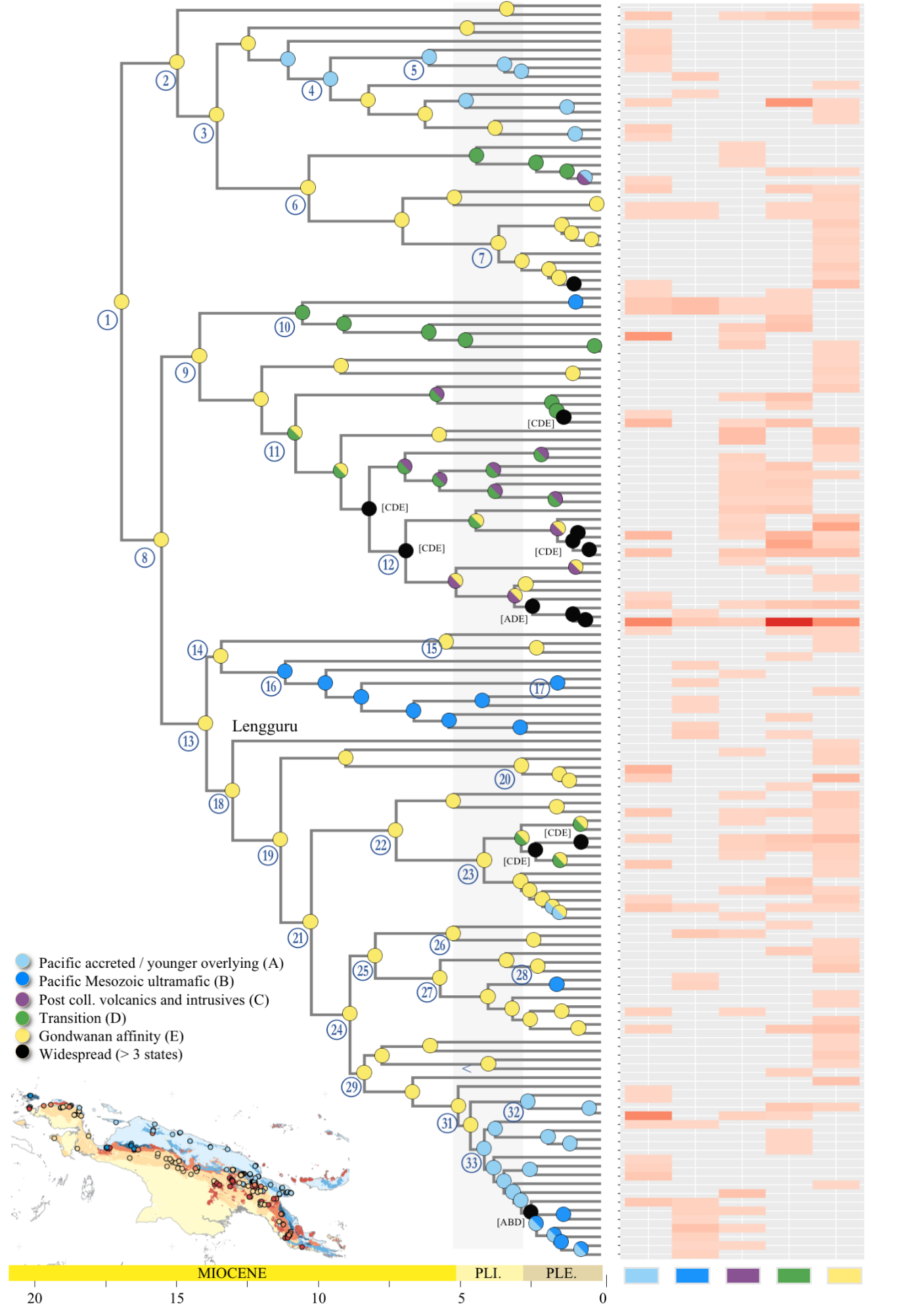


2D histogram (“Heat map”) for distribution of species across geological formations projected onto the phylogenetic hypothesis.


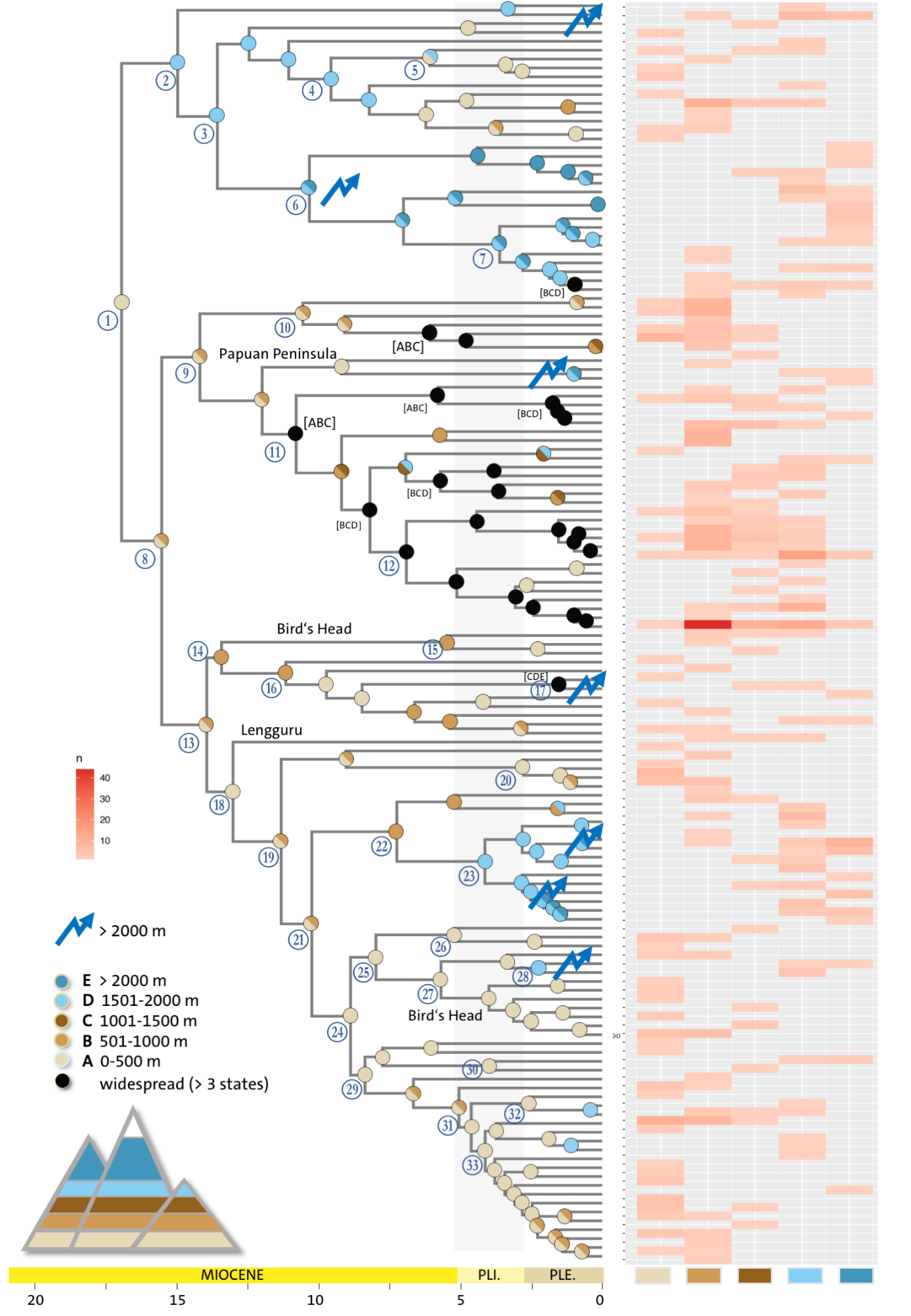


2D histogram (“Heat map”) for distribution of species across elevational bands projected onto the phylogenetic hypothesis.


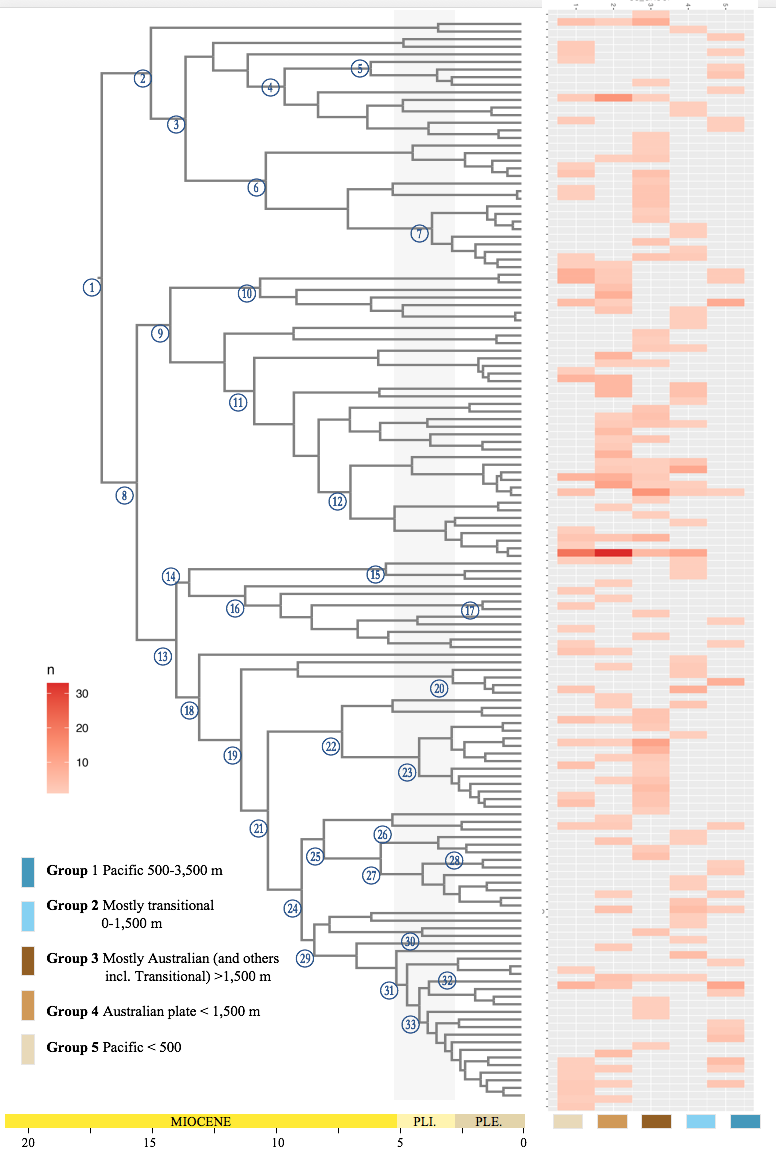


2D histogram (“Heat map”) for distribution of species across five groups as identified by geospatial data analysis, projected onto the phylogenetic hypothesis.

**Appendix 11**

**Detailed outputs of BMM analyses**

Fig. 11A BMM analysis for geology coding, MLS and terminal distribution.

Fig. 11B BMM analysis for geology coding, all states >25% likelihood and terminal distribution.

Fig. 11C BMM analysis for altitude coding, MLS and terminal distribution.

Fig. 11D BMM analysis for altitude coding, all states >25% likelihood and terminal distribution.

Fig. 11E BMM analysis for geography coding, Lengguru area coded as separate entity, MLS and terminal distribution.

Fig. 11F BMM analysis for geography coding, Lengguru area coded as separate entity, all states >25% likelihood and terminal distribution.

Fig. 11G BMM analysis for geography coding, Lengguru area coded as part of the Bird’s Head, MLS and terminal distribution.

Fig. 11H BMM analysis for geography coding, Lengguru area coded as part of the Bird’s Head, all states >25% likelihood and terminal distribution.

**Appendix 12**

Altitudinal distribution map


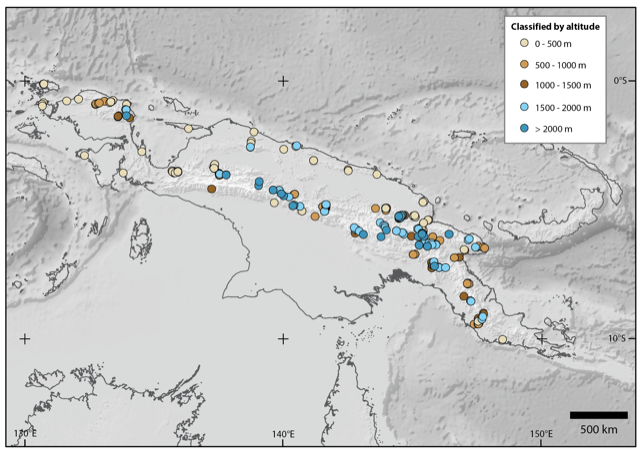


**Appendix 13**

Results of BAMM with a prior of 0.
